# The apple MdCOP1-interacting protein 1 negatively regulates hypocotyl elongation and anthocyanin biosynthesis

**DOI:** 10.1186/s12870-020-02789-3

**Published:** 2021-01-06

**Authors:** Hui Kang, Ting-Ting Zhang, Lu-Lu Fu, Yu-Xin Yao, Chun-Xiang You, Xiao-Fei Wang, Yu-Jin Hao

**Affiliations:** 1 State Key Laboratory of Crop Stress Biology for Arid Areas/Shaanxi Key Laboratory of Apple, College of Horticulture, Northwest A&F University, Yang-Ling, 712100 Shaanxi China; 2 State Key Laboratory of Crop Biology, Shandong Collaborative Innovation Center for Fruit and Vegetable Production with High Quality and Efficiency, College of Horticulture Science and Engineering, Shandong Agricultural University, Tai-An, 271018 Shandong China

**Keywords:** CIP1, COP1, Apple, Hypocotyl, Anthocyanin

## Abstract

**Background:**

In plants, CONSTITUTIVELY PHOTOMORPHOGENIC 1 (COP1) is a key negative regulator in photoperiod response. However, the biological function of COP1-interacting protein 1 (CIP1) and the regulatory mechanism of the CIP1-COP1 interaction are not fully understood.

**Results:**

Here, we identified the apple *MdCIP1* gene based on the Arabidopsis *AtCIP1* gene. Expression pattern analysis showed that *MdCIP1* was constitutively expressed in various tissues of apple, and responded to stress and hormone signals at the transcriptional level. Ectopic expression of *MdCIP1* complemented the phenotypes of the Arabidopsis *cip1* mutant, and MdCIP1 inhibited anthocyanin biosynthesis in apple calli. In addition, the biochemical assay demonstrated that MdCIP1 could interact with MdCOP1 protein by their coiled-coil domain, and *MdCIP1-OX/cop1–4* had a similar phenotype in photomorphogenesis with the *cop1–4* mutant, suggesting that *COP1* is epistatic to *CIP1*. Furthermore, the transient transformation assay indicated that MdCIP1 repressed anthocyanin biosynthesis in an MdCOP1-mediated pathway.

**Conclusion:**

Take together, this study finds that MdCIP1 acts as a repressor in regulating hypocotyl elongation and anthocyanin biosynthesis through MdCOP1 in apple.

## Background

From seed germination to seedling development, plants are precisely regulated by various environmental factors. Light is one of the most important environmental factors affecting plant growth, development and reproduction. Plants convert light energy into chemical energy through photosynthesis and store it in plants. They can also sense light signals through the photoreceptor, and then regulate the entire life cycle of plant growth and development [1, 2]. Plants mainly rely on three kinds of photoreceptors to sense sunlight and transmit light signals, namely phytochromes (red and far-red light receptors), cryptochromes (blue light receptor) and UVR8 (UV-B light receptor) [3–5].

After being activated by light, photoreceptors enter the nucleus and interact directly with a series of factors to transmit light signals, thus changing the transcription pattern of plants, and finally leading to photomorphogenesis [6, 7]. Photomorphogenesis is essential for seedling development. When the seeds germinate in the soil, the seedlings accelerate hypocotyl elongation to emerge from the soil. After reaching the light, hypocotyl elongation is inhibited while cotyledons quickly expand [8]. In addition, the biosynthesis of chlorophyll and anthocyanin is also a typical feature of photomorphogenesis [8, 9]. In the process of light signal transduction, CONSTITUTIVELY PHOTOMORPHOGENIC 1 (COP1) is the most important negative factor, which is located downstream of photoreceptors [10]. COP1 is a conserved RING type E3 ubiquitin ligase, which participates in many physiological processes of different organisms, including plant growth and development, mammalian cell growth and metabolism [11].

In plants, COP1 is transported into the nucleus under dark conditions, thus regulates the seedlings to skotomorphogenesis by degrading the photomorphogenesis promoters LONG HYPOCOTYL IN FAR-RED 1 (HFR1), LONG AFTER FAR-RED LIGHT 1 (LAF1) and ELONGATED HYPOCOTYL 5 (HY5); under light conditions, COP1 is located in the cytoplasm, thus promotes biosynthesis of the downstream substrate, which promotes photomorphogenesis [12–14]. In addition, COP1 is involved in plant flowering, stomatal opening and closing, biological rhythm and fruit development [15–17]. Moreover, COP1 also participates in plant defense response, hormone signaling and miRNA biosynthesis [18–21].

COP1 protein contains an N-terminal RING finger motif, followed by a coiled-coil domain and WD40 repeats, and several COP1-interacting proteins (CIPs) have been isolated in Arabidopsis [22–28]. Among them, CIP4 and CIP7 interact with coiled-coil domain of COP1, which are located in the nucleus and have transcriptional activation activity. CIP4 and CIP7 are also positive regulators of photomorphogenesis and potential direct downstream targets of COP1 [25, 27]. However, the functions of CIP4 and CIP7 are different. The decrease of CIP7 expression resulted in the decrease of light-dependent anthocyanin and chlorophyll biosynthesis, and did not affect the hypocotyl elongation. While the decrease of CIP4 expression inhibited hypocotyl elongation and chlorophyll content, and did not affect anthocyanin biosynthesis [25, 27]. CIP8 is a RING-H2 ligase, which can interact with the COP1 protein. CIP8 can also degrade HY5 through a ubiquitin ligase E2-dependent manner in vitro, but its biological function is not yet clear [26, 28].

Previous studies have found that CIP1 interacts with the coiled-coil domain of COP1 in vitro [24]. But the biological function of CIP1 and the biological significance of the CIP1-COP1 interaction are not fully understood. Here, we found that the apple homologous gene *MdCIP1* negatively regulates photomorphogenesis. Moreover, MdCIP1 interacts with MdCOP1 and inhibits apple anthocyanin biosynthesis in an MdCOP1-mediated pathway.

## Results

### Protein structure of MdCIP1

The apple *MdCIP1* (*MDP0000230486*) gene was blasted from the database Apple Genome V1.0 Predicted Peptides by using the protein sequence of Arabidopsis AtCIP1 (AT5G41790). After SMART analysis of protein secondary structure (http://smart.embl-heidelberg.de/smart/set_mode.cgi?NORMAL=1), MdCIP1 contains a potential coiled-coil domain, corresponding to aa 251–684 of the full length MdCIP1, which is analogous to AtCIP1 (Fig. S1b). Then, Pymol program and VMD software were used to predict the 3D structure of MdCIP1 and AtCIP1. Surprisingly, the 3D structure of MdCIP1 and AtCIP1 is highly similar, although their protein sequence similarity is only 30% (Fig. S1a, c).

### Expression patterns of *MdCIP1*

**Fig. 1:**
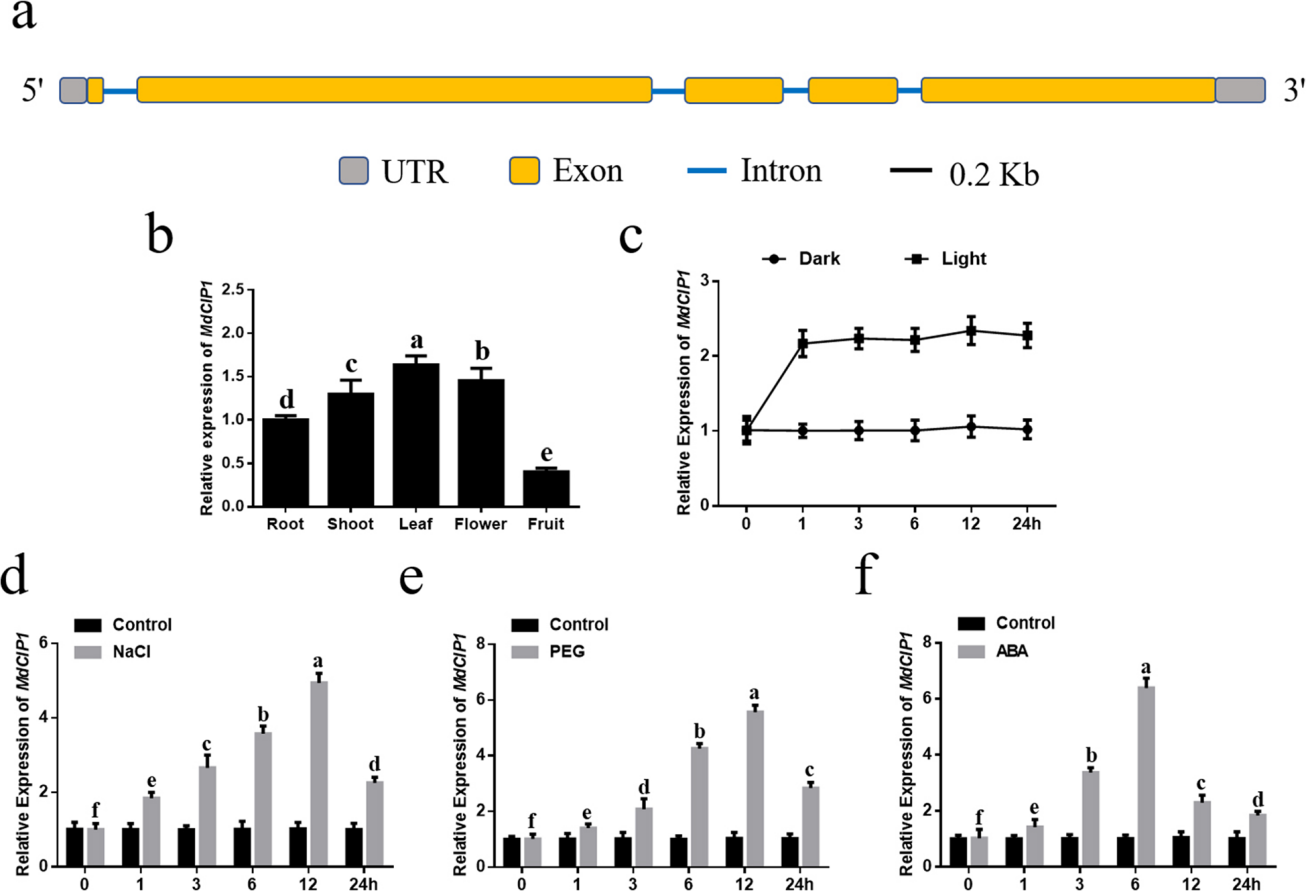
Expression patterns of *MdCIP1*. **a** Schematic diagram of genomic structure of the *MdCIP1* gene. **b** *MdCIP1* transcript level in different apple tissues. The data represented as relative to root. **c** Comparison of relative expression of *MdCIP1* in the leaves of apple cultured seedlings exposed to light and dark treatment at 0, 1, 3, 6, 12 and 24 h. **d-f** Comparison of relative expression of *MdCIP1* in the leaves of apple cultured seedlings between NaCl/PEG/ABA treatment and control at 0, 1, 3, 6, 12 and 24 h. Results represent as means ± SE. Means with different letters indicate statistically significant differences (one-way ANOVA, Tukey-Kramer test, *P* < 0.05)

Through Gene Structure Display Server 2.0 analysis, it was found that the *MdCIP1* gene contains five exons, four introns and encodes 1640 amino acids (Fig. 1a). In order to analyze the tissue-specific expression of *MdCIP1*, the transcription level of *MdCIP1* in root, shoot, leaf, flower and fruit was detected. It was found that the expression level of *MdCIP1* was higher in leaves and lower in fruits (Fig. 1b). The response of *MdCIP1* to light and dark conditions was also analyzed. After 1 h of light treatment, the expression of *MdCIP1* increased rapidly. With the prolongation of the illumination time, the expression of *MdCIP1* did not change significantly. In dark treatment, there was no significant difference in expression level of *MdCIP1* at different time (Fig. 1c). The response of *MdCIP1* to stress and hormone signals was also examined. As a result, in the early stage of salt and PEG treatment, the *MdCIP1*expression was significantly up-regulated, reached the maximum after 12 h treatment, and then decreased (Fig. 1d, e). The response of *MdCIP1* to ABA is faster than salt and PEG treatment, reaching the maximum after 6 h treatment (Fig. 1f). These results indicate that *MdCIP1* can respond to stress and hormone signals at the transcriptional level.

### MdCIP1 inhibits photomorphogenesis

**Fig. 2:**
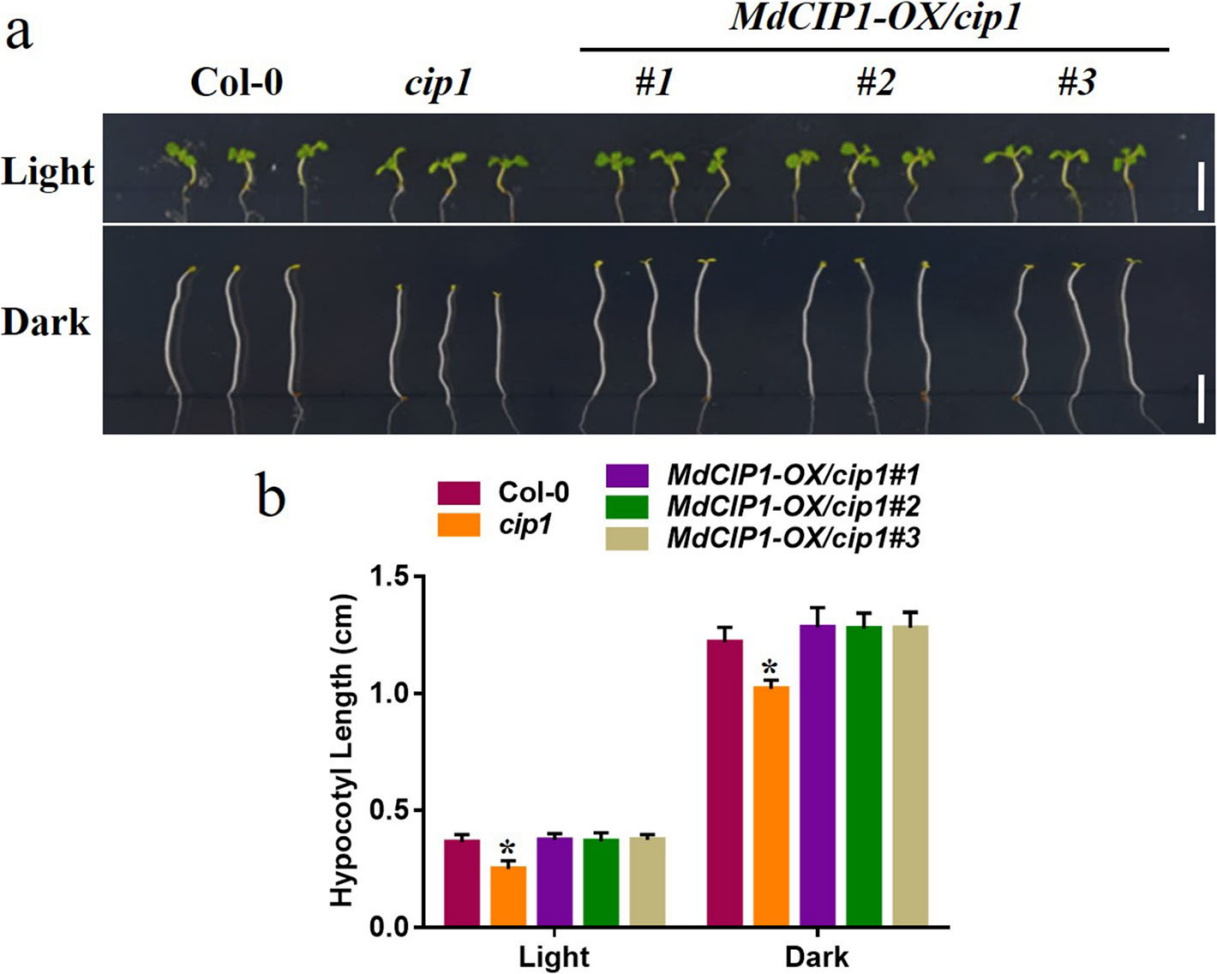
*MdCIP1* inhibits photomorphogenesis. **a-b** Representative picture and measurement of the hypocotyl of col-0, the *cip1* mutant and *MdCIP1-OX/cip1#1/2/3* Arabidopsis seedlings under the dark and light. Three independent biological replicates were performed. Results represent as means ± SE (*n* = 20). Asterisks indicate significantly different values (*P < 0.05). Bar = 0.5 cm

**Fig. 3:**
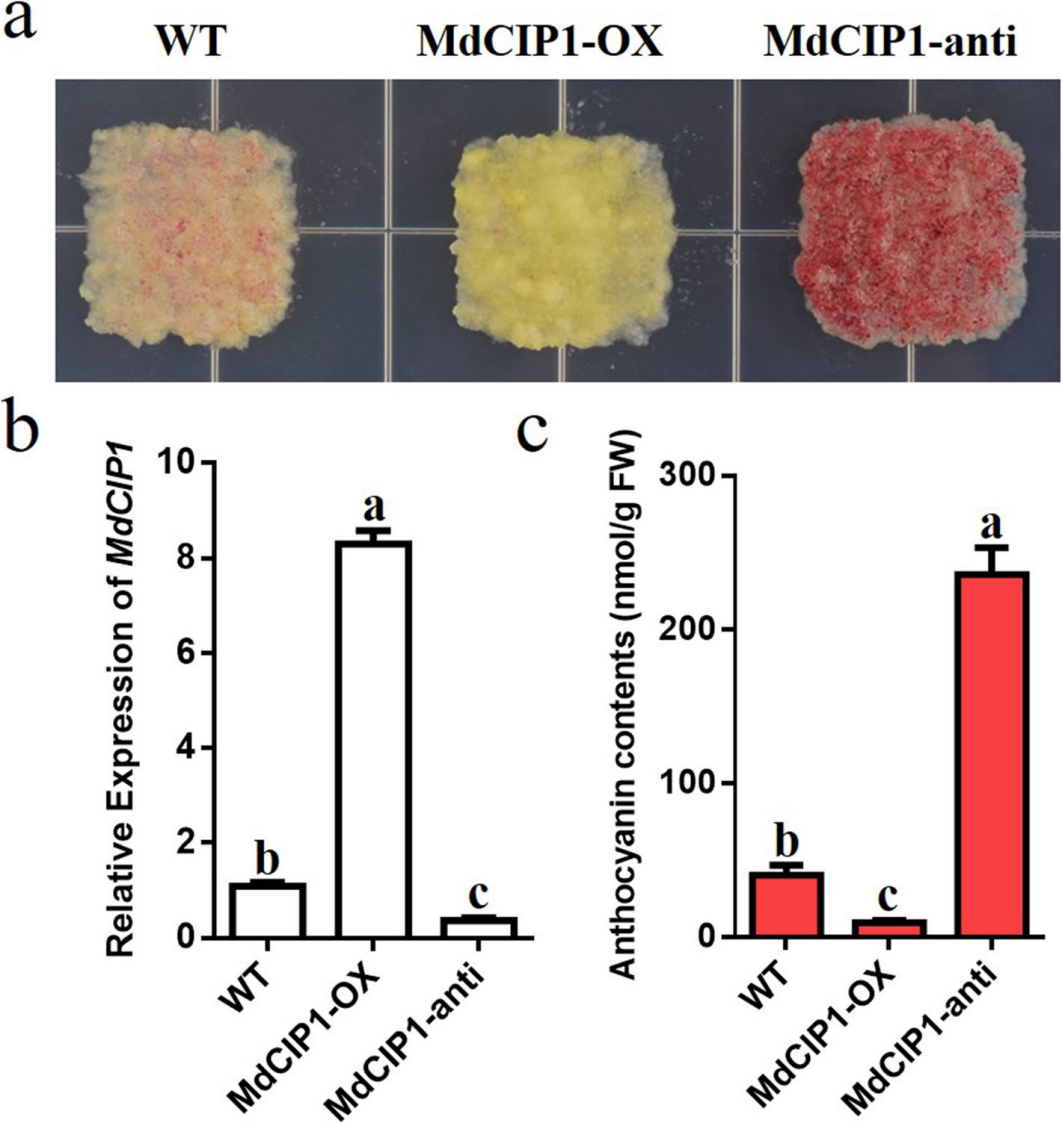
MdCIP1 represses anthocyanin biosynthesis in apple calli. **a** Anthocyanin phenotype of the transgenic MdCIP1-OX and MdCIP1-anti calli. **b** The relative expression level of *MdCIP1* in the transgenic MdCIP1-OX and MdCIP1-anti calli. **c** Anthocyanin content of the transgenic MdCIP1-OX and MdCIP1-anti calli. Results represent as means ± SE, from three independent biological replicates. Means with different letters indicate statistically significant differences (one-way ANOVA, Tukey-Kramer test, P < 0.05)

To investigate the function of CIP1 in photomorphogenesis, the *cip1* mutant, *MdCIP1* heterologous overexpression lines and their hybrid Arabidopsis materials were obtained. These materials were identified at the DNA level (Fig. S2). The hypocotyl length of seedlings in light and darkness was quantified. As a result, the hypocotyl of the *cip1* mutant was shorter than that of wild type under light and dark conditions (Fig. 2) [29]. *MdCIP1* overexpression in the *cip1* mutant complemented the phenotype of the *cip1* mutant (Fig. 2). This result suggests that AtCIP1 and MdCIP1 play an inhibitory role in photomorphogenesis and are conserved in function. To further understand the function of MdCIP1 in apple, the *MdCIP1-OX* and *MdCIP1-anti* transgenic apple calli were constructed (Fig. 3b). The calli anthocyanin biosynthesis results show that compared with WT, MdCIP1-OX accumulated less anthocyanin, and MdCIP1-anti accumulated obviously more (Fig. 3a, c). This suggests that MdCIP1 represses anthocyanin biosynthesis in apple calli.

### MdCIP1 interacts with MdCOP1

**Fig. 4:**
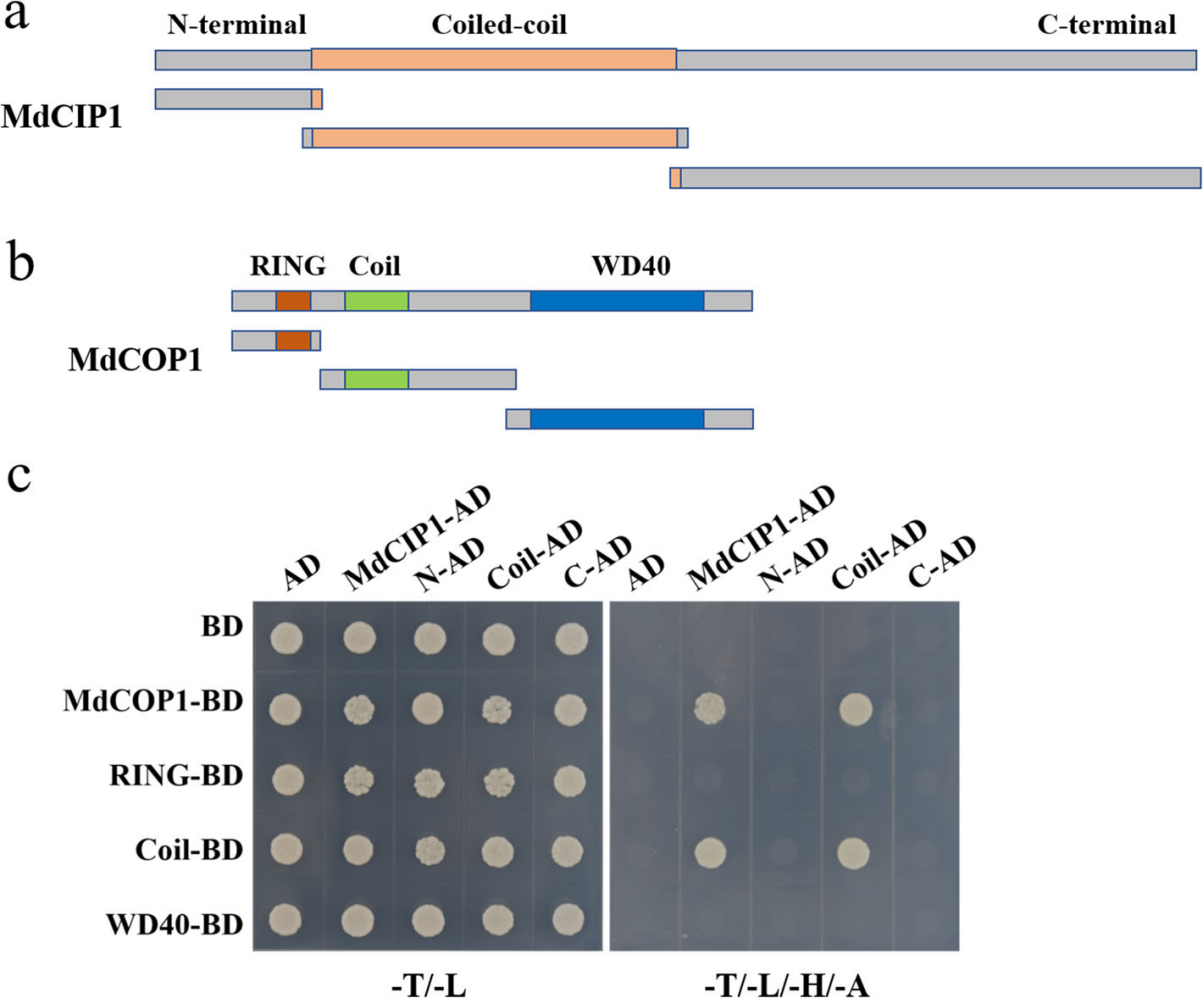
MdCIP1 interacts with MdCOP1 in Y2H assay. **a** Schematic diagram of MdCIP1 protein domains. **b** Schematic diagram of MdCOP1 protein domains. **c** Different truncated segments of *MdCIP1* and *MdCOP1* were connected to AD and BD vectors, respectively. MdCIP1 interacts with the coiled-coil domain of MdCOP1 via its coiled-coil domain

AtCIP1 interacts with AtCOP1 protein by their coiled-coil domains [24]. It is speculated that MdCIP1 interacts with MdCOP1, and was verified by Y2H experiment. According to the protein domain analysis, MdCIP1 is divided into N-terminal, a coiled-coil domain and C-terminal; MdCOP1 contains a RING motif, a coiled-coil domain and WD40 repeats (Fig. 4a, b). Y2H results show that MdCIP1 also interacts with the coiled-coil domain of MdCOP1 via its coiled-coil domain (Fig. 4c). Moreover, the pull-down assay was also used to verify this interaction. The results showed that His-MdCOP1 protein was pulled down only by GST-MdCIP1 CC protein but not GST protein, demonstrating that the coiled-coil domain of MdCIP1 physically interacts with MdCOP1 in vitro (Fig. S3). MdCIP1 also interacts with AtCOP1 (Fig. S4), which is similar to that of AtCIP1-AtCOP1.

### *COP1* is epistatic to *CIP1*

**Fig. 5:**
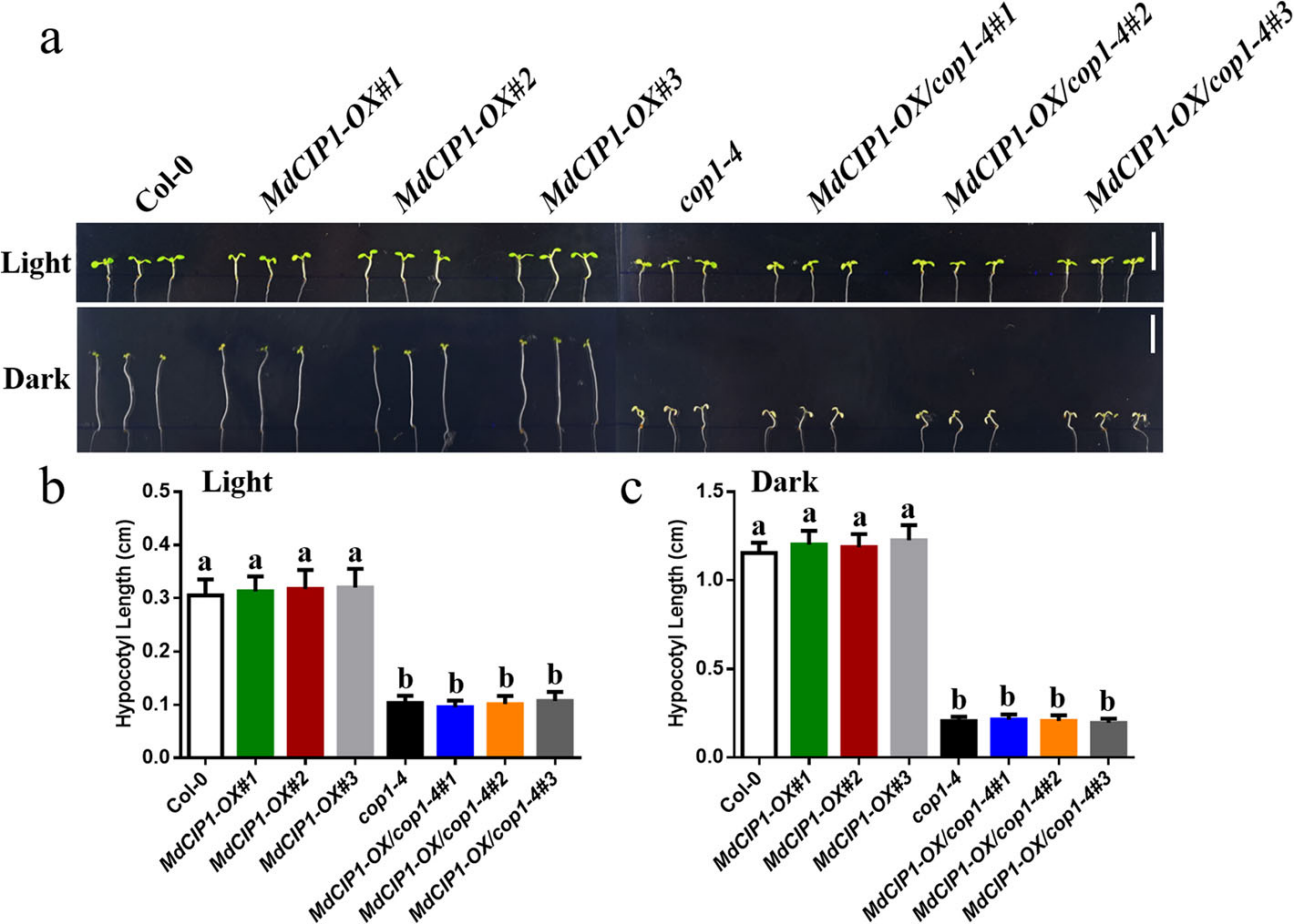
*COP1* is epistatic to *CIP1*. **a-b** Representative picture and measurement of the hypocotyl of col-0, *MdCIP1-OX*, *cop1–4* and *MdCIP1-OX/cop1–4* Arabidopsis seedlings under the dark and light. Three independent biological replicates were performed. Results represent as means ± SE (n = 20). Means with different letters indicate statistically significant differences (one-way ANOVA, Tukey-Kramer test, P < 0.05). Bar = 0.5 cm

In order to test whether *CIP1* is dependent on *COP1* in photomorphogenesis*,* the *cop1–4* mutant, *MdCIP1-OX* and *MdCIP1-OX/cop1–4* were obtained and proven by the DNA detection (Fig. S5). Interestingly, ectopic overexpression of *MdCIP1* in the *cop1–4* mutant showed a similar phenotype of hypocotyl with that of *cop1–4*, no matter the light or dark treatments (Fig. 5). At the adult stage, *MdCIP1-OX/cop1–4* also fully exhibited the features of *cop1–4*, dwarfing and branching (Fig. S6). This result indicates that *COP1* is epistatic to *CIP1*.

### MdCIP1 represses anthocyanin biosynthesis in an MdCOP1-dependent pathway

**Fig. 6:**
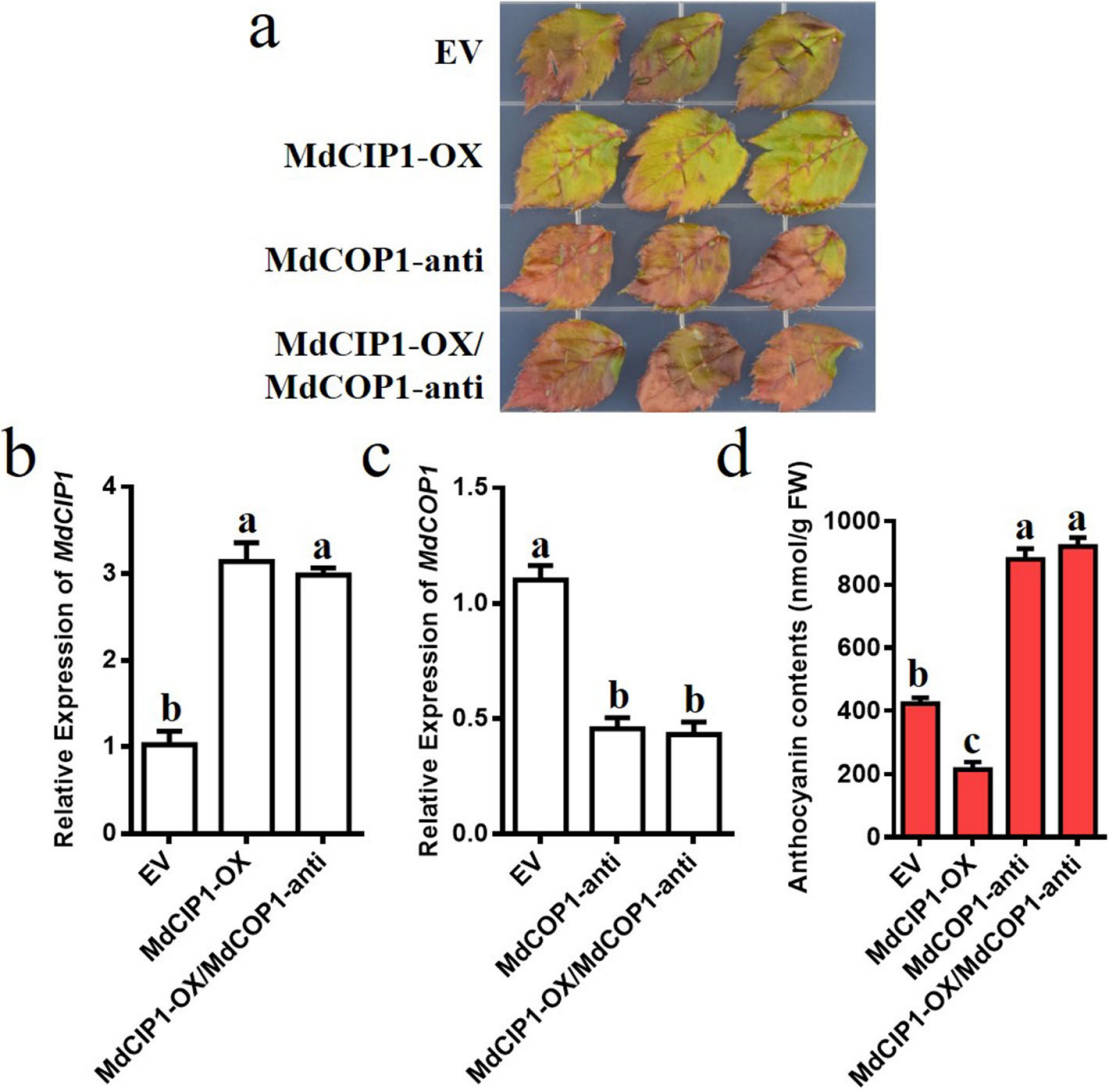
MdCIP1 represses anthocyanin biosynthesis in an MdCOP1-dependent pathway in apple leaves. **a** Representative images of anthocyanin biosynthesis of the apple leaves. **b** Relative expression level of *MdCIP1* after injection of MdCIP1-OX and MdCIP1-OX/MdCOP1-anti. **c** Relative expression level of *MdCOP1* after injection of MdCOP1-anti and MdCIP1-OX/MdCOP1-anti. **d** Anthocyanin content of the apple leaves. Three independent biological replicates were performed. Results represent as means ± SE. Means with different letters indicate statistically significant differences (one-way ANOVA, Tukey-Kramer test, P < 0.05)

The roles of MdCIP1 and MdCOP1 in anthocyanin biosynthesis were also observed by transient infection of leaves. Empty vector served as a control. As a result, the *MdCIP1* expression was up-regulated and anthocyanin decreased after MdCIP1-OX infection; the *MdCOP1* expression was down-regulated and anthocyanin increased after MdCOP1-anti infection (Fig. 6a-d). When mixed infection, the anthocyanin content of MdCIP1-OX/MdCOP1-anti was equal to that of MdCOP1-anti (Fig. 6a-d). This indicates that MdCIP1 represses anthocyanin biosynthesis through MdCOP1 in apple leaves.

## Discussion

`In Arabidopsis, CIP1 encodes 1586 amino acids and its relative molecular weight is up to 182.02 kDa [24]. MdCIP1 protein contains 1640 amino acids with a relative molecular weight of 182.92 kDa. Although the protein sequence similarity of MdCIP1 and AtCIP1 is only 30%, their 3D structures were remarkably similar (Fig. S1). This indicates that the amino acid sequence of CIP1 protein varies greatly with evolution among different species, but the key amino acids that maintain the protein structure remain unchanged. Structure determines function, which further indicates that CIP1 protein may have a conservative function during evolution. Figure 2 showed that both MdCIP1 and AtCIP1 restrain photomorphogenesis, which may prove this point.

*MdCIP1-OX/cop1–4* showed a similar phenotype with that of *cop1–4*, indicating that *CIP1* is located upstream of *COP1* (Fig. 5; Fig. S6). CIP1 could interact with COP1 protein by their coiled-coil regions [24]. It is suggested that CIP1 may regulate COP1 at the protein level. COP1 has three domains: RING domain is responsible for recruiting ubiquitin-binding enzyme E2, the coiled-coil domain for dimerization and COP1-SPA interaction to form COP1-SPA complexes, and WD40 repeat region for interacting with substrates [11, 30]. SPAs interact with COP1 and enhance the E3 ubiquitin ligase activity of COP1 [31–33]. Recently, a novel COP1 interacting protein, COP1 SUPPRESSOR 2 (CSU2) was identified [34]. CSU2 is located in the nucleus and could interact with COP1 through their coiled-coil regions. CSU2 represses COP1 E3 ubiquitin ligase activity by potentially interrupting dimerization of COP1 or COP1-SPA interaction [34]. However, CIP1, as a cytoskeleton-associated protein, exhibits a fibrillar pattern in the cytoplasm in hypocotyl cells [24]. Recent studies have shown that COP1 in the cytoplasm ubiquitously degrades the microtubule-associated protein WAVE-DAMPENED 2-LIKE 3 (WDL3) under darkness in hypocotyl cells [35, 36]. It is speculated that CIP1 may be involved in the degradation of WDL3 by regulating COP1 protein in the cytoplasm, which is different from CSU2.

For apples, anthocyanin is important, which not only determines the color quality to a large extent, but also has the effect of antioxidant and scavenging reactive oxygen [37, 38]. Anthocyanins, a class of key secondary metabolites, are synthesized by a series of enzymes (PAL, CHS, CHI, F3H, DFR, ANS and UFGT) encoded by structural genes and regulated by the MYB-bHLH-WD40 transcription complex [39, 40]. Anthocyanin biosynthesis is affected by environmental and hormonal signals [38, 41]. MdMYB1 and its alleles (MdMYB10 and MdMYBA) promotes anthocyanin biosynthesis by activating the transcription of structural genes *MdDFR* and *MdUF3GT* [42, 43]. Studies have found that MdBT2 accelerated the degradation of MdMYB1 through proteasome pathway, thereby negatively regulating nitrogen-deficient induced anthocyanin biosynthesis [44]. Meanwhile, ABA, wounding, drought stress, and high light also induce anthocyanin biosynthesis through the “MdBT2-Target protein-MdMYB1” module (Target protein: MdbZIP44, MdWRKY40, MdERF38 and MdTCP46, respectively) [45–49]. MdCOP1 negatively regulate anthocyanin biosynthesis and the peel coloration of apple fruits by ubiquitination degradation of the MdMYB1 protein [50]. This study found that MdCIP1 repress anthocyanin biosynthesis, and MdCOP1 participates in this process (Fig. 6). The promoter activity of *AtCIP1* can be induced by osmotic stress and ABA, and AtCIP1 is positively involved in ABA response [29]. *MdCIP1* responds to hormonal and environmental signals at the transcriptional level (Fig. 1). It is presumed that the MdCIP1-MdCOP1-MdMYB1 pathway may also be used to reveal the regulation mechanism of anthocyanin biosynthesis by hormonal and environmental signals, providing a new perspective on the hormonal and environmental impact on fruit coloration.

## Conclusion

MdCIP1, upstream regulator of MdCOP1, plays an inhibitory role in hypocotyl elongation and anthocyanin biosynthesis by interacting with MdCOP1 in apple.

## Methods

### Plant materials and growth conditions

The ‘Orin’ apple calli was provided by Prof. Takaya Moriguchi of the National Institute of Fruit Tree Science, Japan, and has been widely used in peer laboratories [51, 52]. The ‘Orin’ apple calli were cultured on MS medium with 0.4 mg/L 6-BA, 1.5 mg/L 2,4-D and 3% sucrose at 25 °C. The apple calli were subcultured at 3 weeks intervals under darkness.

A line with high regeneration capacity isolated from ‘Royal Gala’ named ‘GL-3’ has been widely used in peer laboratories [53, 54]. We declare that the collection of plant materials complies with institutional, national, or international guidelines. The ‘GL-3’ apple culture seedling was provided by Prof. Zhi-Hong Zhang of Shenyang Agricultural University, China. The GL3 apple culture seedlings were cultured on MS medium with 0.5 mg/L 6-BA, 0.1 mg/L GA_3_, 0.2 mg/L NAA and 3% sucrose under 16 h light/8 h dark condition at 25 °C. The 5-year-old ‘GL-3’ apple trees in the experimental station of Shandong Agricultural University were used for tissue expression analysis. For expression pattern analysis, one-month-old apple culture seedlings were treated with light, darkness, NaCl, PEG and ABA, respectively [55]. The culture seedlings were pretreated in the dark for one day, half of which were treated with light (100 μmol/m^2^/s), and the other half treated with darkness. The culture seedlings were pretreated in liquid medium for one day, and then transferred to liquid medium containing 150 mmol/L NaCl, 10% PEG6000 and 100 μmol/L ABA, respectively. The whole seedlings were sampled at 0, 1, 3, 6, 12, and 24 h after treatment for subsequent analyses. Each treatment was performed with at least three independent biological repetitions.

*Arabidopsis thaliana* ecotype Columbia-0 (Col-0), was used as wild type, obtained from Arabidopsis Biological Resource Center (ABRC). The *cip1* (Salk_070302) mutant was provided by Prof. Qing-Qiu Gong of Nankai University, China, which originally obtained from ABRC [29]. The *cop1–4* mutant was provided by Prof. Hong-Quan Yang of Shanghai Normal University, China [23]. Surface-sterilized Arabidopsis seeds were sown on 1/2 MS medium with 1% sucrose. The seeds were kept at 4 °C for 3 d in the dark, then transferred to a 16 h-light period at 22 °C.

### Vector construction and plant transformation

Leaves of ‘GL-3’ culture seedlings were used to clone the coding sequence of *MdCIP1* and *MdCOP1*. Leaves of Col-0 seedlings were used to clone the coding sequence of *AtCOP1*. To get the transgenic lines, the coding sequence and antisense fragment of *MdCIP1 or MdCOP1* were inserted into pRI101 vector by SalI and BamHI double digestion, and *Agrobacterium tumefaciens* strain GV3101 was used to transform apple calli and Arabidopsis, and *35S::MdCIP1-OX* transgenic Arabidopsis*, 35S::MdCIP1-OX*, *35S::MdCIP1-anti* and *35S::MdCOP1-anti* transgenic calli were obtained, respectively. Transformations of Arabidopsis and apple calli followed the methods described in [56, 57]. The primers for vector construction are shown in Additional file 1: Table S1.

### RNA extraction and qRT-PCR analysis

Total RNA of plant materials was extracted using RNA plant Plus Reagent Kit (CWBIO, China), and PrimeScript first-strand cDNA synthesis kit (Takara, Japan) was used to performed reverse transcription assay. The qRT-PCR was performed using the UltraSYBR Mixture (CWBIO, China) and ran on ABI Step One Plus system (ABI, USA). *Md18S* was used as the reference gene. The qRT-PCR program was performed under the following condition (40 cycles): 95 °C for 15 s and 60 °C for 40s. The qRT-PCR assay was carried out by three independent biological and three technical replicates. 2^-ΔΔCt^ method was applied to calculate relative transcript level [58]. The specific primers are listed in Additional file 1: Table S1.

### Protein sequence alignment and 3D structure prediction

Protein sequence alignment was performed by the DNAMAN software. Protein 3D structure was predicted by Phyre^2^ (http://www.sbg.bio.ic.ac.uk/phyre2/html/page.cgi?id=index), and composite was obtained using the Pymol program v6.5.1. Root-mean-square deviation (RMSD) values were calculated by VMD software v1.2.1 to reflect the match [59]. The genomic sequence of the *MdCIP1* gene was selected and downloaded from Phytozome v12.1 (https://phytozome.jgi.doe.gov/pz/portal.html).

### Yeast two-hybrid (Y2H) assay

Different truncated segments of *MdCIP1* were connected to pGAD424 (AD) by EcoRI and BamHI double digestion. Different truncated segments of *MdCOP1* were connected to pGBT9 (BD) by EcoRI and BamHI double digestion. Different truncated segments of *AtCOP1* were connected to pGBT9 (BD) by EcoRI and SalI double digestion. The Y2H assay was performed according to the Yeast Transformation System 2 protocol (Clontech). The two plasmids were co-transformed into the Y2H strain using the lithium acetate method and cultured at 28 °C. The yeast was grown on yeast selective medium lack of tryptophan and leucine (−T/−L) and then lack of tryptophan, leucine, histidine and Adenine (−T/−L/−H/−A) [60]. The primers are shown in Additional file 1: Table S1.

### Pull-down assay

The full-length sequence of MdCOP1 was introduced into the pET-32a (His) by EcoRI and SalI double digestion. The coiled-coil domain of MdCIP1 was introduced into the pGEX-4 T-1 (GST) by BamHI and SalI double digestion. The recombinant plasmids were transformed into *Escherichia coli* BL21 (TransGen Biotech) to express His-MdCOP1 and GST-MdCIP1 CC protein, respectively. The pull-down assay was performed according to the instructions of the Pierce™ GST Spin Purification Kit (Thermo Fisher Scientific). Then samples were detected by western blotting with anti-His and anti-GST antibodies, respectively.

### Determination of the total anthocyanin content

To accumulate anthocyanin, 15-day apple calli were grown on low-nitrogen (0.2 mmol/L nitrate) medium under strong light supplemented with UV-B for 7 d at 17 °C. Approximately 0.1 g of the samples were soaked and incubated in 1 ml extraction buffer (95% ethanol:1.5 M HCl = 85:15, v/v) overnight in the dark at room temperature. The absorbances were measured at 530, 620 and 650 nm using a spectrophotometer. OD_λ_ = (A_530_ - A_620_) - 0.1 (A_650_ - A_620_). The anthocyanin content was quantified by the following formula: OD_λ_/ξ_λ_ × V/m × 10^6^ (nmol × g^− 1^ fresh weight; V: volume; m: weight; ξ_λ_: 4.62 × 10^4^) [61].

### Measurement of the hypocotyl length

The Arabidopsis material including Col-0, *cip1*, *cop1–4*, *MdCIP1-OX/cip1#1/2/3*, *MdCIP1-OX/cop1–4#1/2/3* were used to quantify the length of hypocotyl. The seeds were kept at 4 °C for 3 d in the dark. Then the seeds were transferred to light (16 h light/8 h dark) or dark conditions, and grown at 22 °C for 5 d. The ImageJ software was used to measure the length of hypocotyls. Approximately 20 hypocotyls were quantified for each material. The experiments were performed with at least three independent biological repetitions.

### Transient transformation in apple leaves

The 35S::MdCIP1-OX (pRI101-MdCIP1), 35S::MdCOP1-anti (pRI101-asMdCOP1) and EV (pRI101) plasmids were transformed into *Agrobacterium tumefaciens* strain GV3101. The leaves cut from the 20-day apple culture seedlings were incubated with *Agrobacterium*, and vacuumed twice for 20 min. The transiently transformed apple leaves were treated on low-nitrogen (0.2 mmol/L nitrate) medium under strong light supplemented with UV-B for 3–5 d at 17 °C. The leaves were harvested for gene expression analysis and anthocyanin content determination [62]. Each experiment required about 60 leaves, and at least three independent biological repetitions were carried out.

## Supplementary Information

**Additional file 1: Table S1.** Primers used in this study.

**Additional file 2: Table S1–4.** The data of hypocotyl length and anthocyanin content presented in this study.

**Additional file 3.** The coding and protein sequences of MdCIP1, MdCOP1, AtCIP1 and AtCOP1.

**Additional file 4.** The original gel/blot images presented in this study.

**Additional file 5: Fig. S1.** The protein structure comparison of MdCIP1 and AtCIP1. **Fig. S2.** Identification of the *MdCIP1-OX/cip1* Arabidopsis seedlings at the DNA level. **Fig. S3.** The coiled-coil region of MdCIP1 interacts with MdCOP1 in vitro pull-down assay. **Fig. S4.** MdCIP1 interacts with AtCOP1. **Fig. S5.** Identification of the *MdCIP1-OX/cop1–4* Arabidopsis seedlings at the DNA level. **Fig. S6.** *MdCIP1-OX/cop1–4* presents the *cop1–4* phenotype at the adult stage.

## Data Availability

All the data about the present study has been included in the table and/or figure form in the current manuscript or additional files. The MdCIP1 (MDP0000230486) and MdCOP1 (MDP0000245133) sequences are available at apple genome database V1.0 (https://www.rosaceae.org/) [50]; the AtCIP1 (AT5G41790) and AtCOP1 (AT2G32950) sequences are available at Arabidopsis database (https://www.arabidopsis.org/).

## Abbreviations

COP1: Constitutively photomorphogenic 1
LAF1: Long after far-red light 1;
HY5: Elongated hypocotyl 5
HFR1: Long hypocotyl in far-red 1
CIP: COP1-interacting protein
SPA: Suppressor of phya-105
WDL3: Wave-dampened 2-like 3
CSU2: COP1 suppressor 2
PAL: Phenylalanine ammonialyase
CHI: Chalcone isomerase
CHS: Chalcone synthase
DFR: Dihydroflavonol 4-reductase
F3H: Flavanone 3-hydroxylase
UFGT: Flavonoid 3-O-glucosyltransferase
ANS: Anthocyanidin synthase
Y2H: Yeast two-hybrid
SMART: Simple modular architecture research tool
ABRC: Arabidopsis biological resource center
