# Supplementary material for: The apple MdCOP1-interacting protein 1 negatively regulates hypocotyl elongation and anthocyanin biosynthesis
Source: BMC Plant Biol. 2021 Jan 6;21:15. doi: 10.1186/s12870-020-02789-3 (PMC7789773; doi:10.1186/s12870-020-02789-3)
Supplement: Supplementary file 1 — Additional file 1: Table S1. Primers used in this study. [file 12870_2020_2789_MOESM1_ESM.docx]

**Table S1** Primers used in this study

| **Name** | **Primer (5′-3′)** | |  |
| --- | --- | --- | --- |
| Md18S(qRT)-F | | GGGTTCGATTCCGGAGAGG | |
| Md18S(qRT)-R | CCGTGTCAGGATTGGGTAAT | |  |
| AtACTIN2-F | ATCAGCCGTTTTGAATCTCC | |  |
| AtACTIN2-R | CAATCTAACTTCAACAGTTC | |  |
| MdCIP1(qRT)-F | | CATTGGTGCTTGGAGAGC | |
| MdCIP1(qRT)-R | ACAACTGTCTTATGGCTTCC | |  |
| MdCOP1(qRT)-F | AGTAGAGAGATGCAGAAT | |  |
| MdCOP1(qRT)-R | CATTCGACTGTACTGTGT | |  |
| MdCIP1-F | ttgatacatatgcccgtcgacATGCCAAAGCACCGCATG | |  |
| MdCIP1-R | gttgattcagaattcggatccTCAAGGACGAGATGTCGAAGC | |  |
| MdCIP1(anti)-F | GGATCCCAGGCTTTGAGCTGGAGATGG | |  |
| MdCIP1(anti)-R | GTCGACCACTTGTAGCGGTCAATCTCCTC | |  |
| (Y2H) MdCIP1-F | ccaaaaaaagagatcgaattcATGCCAAAGCACCGCATG | |  |
| (Y2H) MdCIP1-R | tctctgcaggtcgacggatccTCAAGGACGAGATGTCGAAGC | |  |
| (Y2H) MdCIP1 N-R | tctctgcaggtcgacggatccGCCGATCAATCACGGTTCTTA | |  |
| (Y2H) MdCIP1 C-F | ccaaaaaaagagatcgaattcCTGTGGCAGGGCTGGAACT | |  |
| (Y2H) MdCIP1 Coil-F | ccaaaaaaagagatcgaattcACAGCGGAATTAAGAACCGTGA | |  |
| (Y2H) MdCIP1 Coil-R | tctctgcaggtcgacggatccTCTGAGCTCCAGTTCCAGCC | |  |
| (Y2H) MdCOP1-F | GAATTCATGCCTGCAGGTCGACGA | |  |
| (Y2H) MdCOP1-R | GGATCCCTATGCCGCAAGAACCAAC | |  |
| (Y2H) MdCOP1 RING-R | GGATCCCTTCTTCAGAAGCTTGTCCAG | |  |
| (Y2H) MdCOP1 Coil-F | GAATTCACTTCTGCTCGTCAAATT | |  |
| (Y2H) MdCOP1 Coil-R | GGATCCGCAGGTAACACTCTTGG | |  |
| (Y2H) MdCOP1 WD40-F | GAATTCCTATTTCACTCAGCCAATAT | |  |
| (Y2H) AtCOP1-F | GAATTCATGGAAGAGATTTCGACG | |  |
| (Y2H) AtCOP1-R | GTCGACTCACGCAGCGAGTACCAG | |  |
| (Y2H) AtCOP1 RING-R | GTCGACCACATGCCGAGCTGAAGT | |  |
| (Y2H) AtCOP1 Coil-F | GAATTCACTTCAGCTCGGCATGTG | |  |
| (Y2H) AtCOP1 Coil-R | GTCGACCGAATCTGACCCACTCAG | |  |
| (Y2H) AtCOP1 WD40-F | GAATTCCAGTCAACTGTCTCAATG | |  |
| *cop1-4*-F | ATGCCGTTGAGAGACATAGAATAG | |  |
| *cop1-4*-R | TCCGTAAGAAGATTCATCCTCTAC | |  |
| *cip1*-LP | TACAGGGAAACACGAAAAACC | |  |
| *cip1*-RP | TCAACCATTCCCAAGATTTTG | |  |
| MdCOP1(anti)-F | GTCGACAATGACCCTCATTCGACTGT | |  |
| MdCOP1(anti)-R | GGATCCGTAGAGAGACGTGGAATGGA | |  |
| MdCOP1 His-F | GAATTCATGGAGGAGTGCTCGACC | |  |
| MdCOP1 His-R | GTCGACTGCCGCAAGAACCAACAC | |  |
| MdCIP1 CC-GST-F | gatctggttccgcgtggatccATGACAGCGGAATTAAGAACCG | |  |
| MdCIP1 CC-GST-R | gatgcggccgctcgagtcgacTCTGAGCTCCAGTTCCAGCC | |  |
