# Supplementary material for: The apple MdCOP1-interacting protein 1 negatively regulates hypocotyl elongation and anthocyanin biosynthesis
Source: BMC Plant Biol. 2021 Jan 6;21:15. doi: 10.1186/s12870-020-02789-3 (PMC7789773; doi:10.1186/s12870-020-02789-3)
Supplement: Supplementary file 2 — Additional file 2: Table S1–4. The data of hypocotyl length and anthocyanin content presented in this study. [file 12870_2020_2789_MOESM2_ESM.docx]

**Table S1** The hypocotyl length of col-0, cip1 and *MdCIP1-OX/cip1#1/2/3* Arabidopsis seedlings in light and dark conditions. ( :cm)

**Table S2** The anthocyanin content in WT, MdCIP1-OX and MdCIP1-anti apple calli. ( :nmol/g FW)

**Table S3** The hypocotyl length of col-0, *MdCIP1-OX#1/2/3*, *cop1-4* and *MdCIP1-OX/cop1-4#1/2/3* Arabidopsis seedlings in light and dark conditions. ( :cm)

**Table S4** The anthocyanin content in the EV, MdCIP1-OX, MdCOP1-anti and MdCIP1-OX/MdCOP1-anti transiently transformed apple leaves. ( :nmol/g FW)
