# Supplementary material for: The apple MdCOP1-interacting protein 1 negatively regulates hypocotyl elongation and anthocyanin biosynthesis
Source: BMC Plant Biol. 2021 Jan 6;21:15. doi: 10.1186/s12870-020-02789-3 (PMC7789773; doi:10.1186/s12870-020-02789-3)
Supplement: Supplementary file 3 — Additional file 3. The coding and protein sequences of MdCIP1, MdCOP1, AtCIP1 and AtCOP1. [file 12870_2020_2789_MOESM3_ESM.docx]

The genomic sequence of *MdCIP1*

ATGCCAAAGCACCGCATGAGGGAAATTAAGTCCTTATTTGGAAGTCACATCAATTCACAGAAGCATGAAGAGCTGAAAGGAACTAAAATAGGTAAATTTTCCTGTAAAAATTCTGCATTAGGCTTTGAGATGGCATTGCAGCACTATACAATGCAACTGGGATAAACTTCACTATGATTACAAATTTCGGTGGATCGATGAAATGGAAATTTAAAGAAGTTTTCTATTTTTACTGCAGGTATTGAGGAGAAGGTGAATAAAATATTGAAGCTTCTAAAAAATGAAGAGCTTAAAAAAGATGACATCCCAGTAGAGAACTCCAAAGTGGAACCTCTCGCTGAGCTAATTCAGGATTTCCACAAGGATTACCAATCACTGTATGCACAATATGATCAACTAACTGGAGCGCTGAAGGAAAAAGTTTGGAGCAAACAAGAAAAGGACAACAGCTCTTCATCAAGTTCAGACTCAGATTCCGATCATTCTTCAAATGAAAAAAGCCGTAAAAATGGAGTGATGGAAAGTGACTTTCAGAAAATAACTGATGGGATCAAGCAGGAACTTGAATTGGCACATCAAGAAGTTGCTGACCTGAAGAGGAGATTGACCGCTACAAGTGAAGAGAAGGAAGCTTTAAGCTCAGATTGTGCGGCGGCTTTGACCAAGATAGAAGAAAAGGAGAGTGTTTTTATTGACTTGAAGACTGCAGCTGAAAGGTTAGATGCTGAAAAATCACTACTTTTGGCTGAGAATGGTGAGCTAAAACAAAAACTGGAAGCTGGTGGAAAGATAGAAGCTGAATTGATTCAAAAAGTGGAAGATGTGGAAGGAGAGAAAGATAACTTGATTAAGGAGAAAGAGACTGCTCTGAGAACGATTGAAGATGGAGAGACTATTACAGCGGAATTAAGAACCGTGATTGATCGGCTGAAAGATGAAAAAGTAACCCTTGAGCAAGAACTAGAATCTGTTCGAGGGGAAGTCATCCATATAAAGCAGAAGCTGCAATCTGCAGAAGAGCAGGTATCAGATTTAAGCCACAATCTGAAAGCCAAGGAGGAAGAAACAACTGAATCTAGTCAGCTACGGGAGAAATTGGGTCAGAGGGAAGTGGAATATTTGGCTCTGTCTGAGATGCATGAGCTGCATGAGAAGGAAACTTTGGCTCAAATTATGGAATTACAGGCTGCTGTGACAGGGCTGGAACTGGAGCTGGAATCTTTGCGAGCTCAGAAAAGAGATATGGAAGTGAAGATTGAGAGCACAGAAGCTGAAGTAAAACAACTGGGAGAGGTGAGTGCAGGACTGCAAGTCCAAATTTCAGAAGTTGAGTCAATATCAAATGAGAAAGCAGCAGAACTTTCTGCTCTCACGAAAAACCTTGAAGATTACAAAAGTGAATCTATTCAGCTGAAGGAGAAATTGGGTCAGAGGGAAGTGGAATGCTCAGCTCTGTCTGAGATGCATGAGCTGCATAAGAGTAAAACATTGGCTCAAATTACGGGATTAGAGGCTGCTGTGGCAGGGCTGGAACTGGAGCTGGAATCTTTGCGAGGTCAGAAAACAGATATAGAAGTTGAGATTGAGAACAAAGAAACTCAAGTGAAACAACTAGCAGAAGAGAACGCGGGACTGCAAGCCCGAATTTCACAACTTGAATCAATATCAAATGAGAGAGAAGCCGAATTTTCTTCCCTGGCAAAGAAATTTGAGGACAGCAGTAATGAATATGGTCAGCTACAGGAGAAACTGGGTCAGAGGGAAGTGGAACACTCAACTCTGTCTGAGATGCATGAGCTGCATAAGAGTAAAACATTGGCTCAAATTACAGGTTTAGAGGCTGCTGTGGCAGGGCTGGAACTGGAGCTGGAATCTTTGCGAGATCAGAAAAAAGATATAGAAGTTGAGATTGAGAAGAAAGAAACTCAAGTGAAACAACTAGCCGAAGAGAATGCGGGATTGCAAGCCCAAACTTCAGAACTTGAATCAATATCAAATGAGAGAGAAGCTGAACMTTCTGCTCTCACCAKAAAACTTGYGGAGATCAATYGTGAATCTATTCAGCWGAAGGAGCAATTGGATCAGAAGGAAAAGGAATACTCAGCTCTGTCTGAGAAGCATGAGCTGCATGAGAGTAAAACATCGGCTCAAATCCTGGGATTAGAGGCTGCTGTGGCAGGGCTGGAACTGGAGCTYAGATCTTTGCGAGGTCAGAAAGAAGATATAGATGTTGAGATTGAGAACAAAGAAACTCAAGTKAAACAACTAGCAGAAGAGAATGTGGGACTGCAAGCCCGAATTTCACAACTTGAATCAATATCAAATGAGAGAGAAGCCGAATTTTCTTCCCTGGKGAAGAAATTTGAGGACAGCAGTAATGAATATGGTCGGCTYCAGGAAAAACTGGGTCAGAGGGAAGTGGAACACTCAACTCTGTCTGAGATGCATGAGCTGCATAAYAGTAAAACTTTGGCTCAAATTACAGGTTTAGAGGCTGCTGTGGCAGGGCTGGAACTGGAGCTGGAATCTTTGKGAGYTCAGAAAAAAGATATAGAAGTTGAGATTGAGAAGAAAGAAACTCAAGTGAAACAACTAGCKGAAGAMAATGCGGGATTGCAAGCCCAAACTTCAGAACTTGTATCAATATCAAATGAGAGAGAAGCTGAACMTTCTGCTCTCACCAKAAAACTTGAGGAGATCAATAGTGAATCTATTCAGCTGAAGGAGKAATTGGYTCAGAYGGAAYWGGAATACTCAGCTCTGTCTGAGAWGCATGAGCTGCATYAGAGTAAAACATRMGCTCAAATRKRGGGATTAGAGGCTGCTGTGGCAGGGCTGGAACTGGAGCTGYYATCTTTGCGAGGTCAGAAAYKAGATATKGAWGTTGAGATTGAGAACAAAGAAACTCAAGTYAAACAACTAGCAGAAGAGAATGRGGGACTGCAAGCCCGAATTTCACAACTTGAATCAATATCAAATGAGAGAGAAGCCGAATTTTCTTCCCTGGCYAAGAAATTTGAGGACAGCAGTAATGAATATGGTCYGCTYCAGGAAAAACTGGGTCAGAMGGAAGTGGAACACTCAACTCTGMCTGAGATGCATGAGCTGCATAAGAGTAAAACMTTGGCTCAAATTACAGGTTTAGAGGCTGCTGTGGCAGGGCTGGAACTGGAGCTGGAATCRTTGKGAGYTCAGAAAYAAGATATAGAYGTTGAGATTGAGAAGAAAGAAACTCAAGTGAAACAACTAGCKGAAGAMAATGRGGGATTGCAAGCCCYAARTTCAGAACTTGWATCAATATCAAATGAGAGAGAAGCTGAACTTTCTGCTCTSACSAAYAAACTTGAGGASAMCAYTAYTGAATKTYMTCAGCTYKAYGAGAAARTGGGTCAGAGGGAAYAGGAATACTCAACTCTGTCTGAGATGCARYAMCTGCATYAGAMTYAAACARTMGKTKAAATWAKYGGATTWGASMCAGKWGTMYCAGGGCTAGKACTGGAGCTGGAATCTTTGCGACATGAGAAYAGWGYTWTSGAAGTAGAGATCGAGAYCAAAGAAACTGTAGCAAAACAACTGGGAGAGGAAAATGCGGGACTGCAAGCCCGAATTTCAGAACTTGAATCGACTTTGAAAGACAGAGAAGCTGAACTTTCCGATCTCACGAAGAAACTTGAGGACAGCAATCATGAATCGTCATCCAGAATAGCAGATTTGTCCGCACAGATCAATAATCTGCTAGCTGAGGTAGATTCTTTGCGTGCCCAGAAAGTTGAGTTGGAGGAACTTATAGTATCCAAAGGTGATGAAGCATCGACTCAGGTCAAGGGATTAACGGAACAAGTAAATGTGTTGCAGCAGGAATTGTTGTCAATGCAGAGCGGGAAAACTGAATTGCAAGTGCAGCTTGAGAACAAAACTCAAGAAGTTTCTGAATTCTTGATACAGATACAGAATCTGAAAGAAGAAATAAACAAACAAGATAACGGATCATGAGAGGGTTGTGGAAGAGAAAGAGAGTTTGACGGCAGAAAAGAGAGAGATTGAGATAAAGGTGGACTCAATACACAACCACAAAAGTGAACTCGAAGAGGAGATAAGAACAAAATGCCTCGAGAGTGATCAATTGAGAGTGGAAATTGTTGAGCTAAGGGATCAAATTGTTGAATTTGAGAGGAGACTAACAGAAAAAGAGGCCGAGTTTTCTTCCCTCCAGGAGAAACATGACAGTGCAGTGAATGACACTTCTGCTCAAATAACAGCCTTTGTATCACAGGTTACTAATCTACAACAGGATTTGGATTCATTGCAGGCTGAGAAGAACCAGATGGAGTTGCAGTTTGAGAGGGAGAAACAAGAACTTTCACAGAGCCTGACGCAATTGGAAAATGAAAAGGTTGAGTTAGAGAGCAAGATTGCTGATCATCAGAGACTGCTGAATGAACACGAGGAGACATATGGCAAGTTAAAAGATGAATATATGCAGCTGGAGAGTCATATCCAGGACAGTAAGGTCAACCAAGACGCTGCAGAAAGGAAAATCGAGCAAATGGCGGAAGATTTCAGTAAGAAAATTGAATCCAAAGATGAGACAATAGCTGATTTGGAGCAAGAGGCTGAATATCTGAAACGAGATCTTGAAGAAAAAGGGTACGAGCTTAGTTCTTTGGTTGAAAACTCCCGTAATGTTGAAGTTAAGCTCCGCCTGTCAAACCAGAAGCTCCGGGTTACAGAGCAGGTATTGACTGAGAAAGAGGAGAGCTTCAGAAAGGCAGAACTGAAATTCCTGGAAGAACAGAGAGCACTTGAAGACAGGATTGCTAGATTGTCTGATATAATCTCTGCAAACAACGAAGCCTATCAAAGAAACATCACACTTGTTGCAGAAAATGTGAACAGTTATTTCAGCGGAATGGAATCCATGATAAAAAAATTTATGGACGACTGTGCAGAGTATGAGACGTGCATTCTGGAAACATCGCAGGAGCTTCACGTTGCAAAGAATTGGGTTGCAGAAACAAGGAGTGAAAGAGAGACGTTGAAGAGGGAGGTGGGGGACCTAATTGAACAACTGCGAGATAAGAAAGAAGAAGCATTGGTGCTTGGAGAGCAGGTCGAGAGGATGCGGGCAAAGGCAAGCAAGGAAGAAGTGGAGAAGGGGAGTCTGATCAAGGCCATGAGCCAACTTGAGAAGAAAGCGGCGGACTTGGAGAAGGTGGTGGAGGAGAAAACAGAGGGAATGCTGGGATTAGGAGAGGAGAAGAGGGAAGCCATAAGACAGTTGTGCATATGGATCGAGTATCACCAAAGCCGGTACGATCATCTCAAGGAAATTCTCTCGAAGACAACTCCTGCAAGAGGCCAGACGAGGGCTTCGACATCTCGTCCTTGA

The coding sequence of *MdCIP1*

ATGCCAAAGCACCGCATGAGGGAAATTAAGTCCTTATTTGGAAGTCACATCAATTCACAGAAGCATGAAGAGCTGAAAGGAACTAAAATAGGTATTGAGGAGAAGGTGAATAAAATATTGAAGCTTCTAAAAAATGAAGAGCTTAAAAAAGATGACATCCCAGTAGAGAACTCCAAAGTGGAACCTCTCGCTGAGCTAATTCAGGATTTCCACAAGGATTACCAATCACTGTATGCACAATATGATCAACTAACTGGAGCGCTGAAGGAAAAAGTTTGGAGCAAACAAGAAAAGGACAACAGCTCTTCATCAAGTTCAGACTCAGATTCCGATCATTCTTCAAATGAAAAAAGCCGTAAAAATGGAGTGATGGAAAGTGACTTTCAGAAAATAACTGATGGGATCAAGCAGGAACTTGAATTGGCACATCAAGAAGTTGCTGACCTGAAGAGGAGATTGACCGCTACAAGTGAAGAGAAGGAAGCTTTAAGCTCAGATTGTGCGGCGGCTTTGACCAAGATAGAAGAAAAGGAGAGTGTTTTTATTGACTTGAAGACTGCAGCTGAAAGGTTAGATGCTGAAAAATCACTACTTTTGGCTGAGAATGGTGAGCTAAAACAAAAACTGGAAGCTGGTGGAAAGATAGAAGCTGAATTGATTCAAAAAGTGGAAGATGTGGAAGGAGAGAAAGATAACTTGATTAAGGAGAAAGAGACTGCTCTGAGAACGATTGAAGATGGAGAGACTATTACAGCGGAATTAAGAACCGTGATTGATCGGCTGAAAGATGAAAAAGTAACCCTTGAGCAAGAACTAGAATCTGTTCGAGGGGAAGTCATCCATATAAAGCAGAAGCTGCAATCTGCAGAAGAGCAGGTATCAGATTTAAGCCACAATCTGAAAGCCAAGGAGGAAGAAACAACTGAATCTAGTCAGCTACGGGAGAAATTGGGTCAGAGGGAAGTGGAATATTTGGCTCTGTCTGAGATGCATGAGCTGCATGAGAAGGAAACTTTGGCTCAAATTATGGAATTACAGGCTGCTGTGACAGGGCTGGAACTGGAGCTGGAATCTTTGCGAGCTCAGAAAAGAGATATGGAAGTGAAGATTGAGAGCACAGAAGCTGAAGTAAAACAACTGGGAGAGGTGAGTGCAGGACTGCAAGTCCAAATTTCAGAAGTTGAGTCAATATCAAATGAGAAAGCAGCAGAACTTTCTGCTCTCACGAAAAACCTTGAAGATTACAAAAGTGAATCTATTCAGCTGAAGGAGAAATTGGGTCAGAGGGAAGTGGAATGCTCAGCTCTGTCTGAGATGCATGAGCTGCATAAGAGTAAAACATTGGCTCAAATTACGGGATTAGAGGCTGCTGTGGCAGGGCTGGAACTGGAGCTGGAATCTTTGCGAGGTCAGAAAACAGATATAGAAGTTGAGATTGAGAACAAAGAAACTCAAGTGAAACAACTAGCAGAAGAGAACGCGGGACTGCAAGCCCGAATTTCACAACTTGAATCAATATCAAATGAGAGAGAAGCCGAATTTTCTTCCCTGGCAAAGAAATTTGAGGACAGCAGTAATGAATATGGTCAGCTACAGGAGAAACTGGGTCAGAGGGAAGTGGAACACTCAACTCTGTCTGAGATGCATGAGCTGCATAAGAGTAAAACATTGGCTCAAATTACAGGTTTAGAGGCTGCTGTGGCAGGGCTGGAACTGGAGCTGGAATCTTTGCGAGATCAGAAAAAAGATATAGAAGTTGAGATTGAGAAGAAAGAAACTCAAGTGAAACAACTAGCCGAAGAGAATGCGGGATTGCAAGCCCAAACTTCAGAACTTGAATCAATATCAAATGAGAGAGAAGCTGAACMTTCTGCTCTCACCAKAAAACTTGYGGAGATCAATYGTGAATCTATTCAGCWGAAGGAGCAATTGGATCAGAAGGAAAAGGAATACTCAGCTCTGTCTGAGAAGCATGAGCTGCATGAGAGTAAAACATCGGCTCAAATCCTGGGATTAGAGGCTGCTGTGGCAGGGCTGGAACTGGAGCTYAGATCTTTGCGAGGTCAGAAAGAAGATATAGATGTTGAGATTGAGAACAAAGAAACTCAAGTKAAACAACTAGCAGAAGAGAATGTGGGACTGCAAGCCCGAATTTCACAACTTGAATCAATATCAAATGAGAGAGAAGCCGAATTTTCTTCCCTGGKGAAGAAATTTGAGGACAGCAGTAATGAATATGGTCGGCTYCAGGAAAAACTGGGTCAGAGGGAAGTGGAACACTCAACTCTGTCTGAGATGCATGAGCTGCATAAYAGTAAAACTTTGGCTCAAATTACAGGTTTAGAGGCTGCTGTGGCAGGGCTGGAACTGGAGCTGGAATCTTTGKGAGYTCAGAAAAAAGATATAGAAGTTGAGATTGAGAAGAAAGAAACTCAAGTGAAACAACTAGCKGAAGAMAATGCGGGATTGCAAGCCCAAACTTCAGAACTTGTATCAATATCAAATGAGAGAGAAGCTGAACMTTCTGCTCTCACCAKAAAACTTGAGGAGATCAATAGTGAATCTATTCAGCTGAAGGAGKAATTGGATATKGAWGTTGAGATTGAGAACAAAGAAACTCAAGTYAAACAACTAGCAGAAGAGAATGRGGGACTGCAAGCCCGAATTTCACAACTTGAATCAATATCAAATGAGAGAGAAGCCGAATTTTCTTCCCTGGCYAAGAAATTTGAGGACAGCAGTAATGAATATGGTCYGCTYCAGGAAAAACTGGGTCAGAMGGAAGTGGAACACTCAACTCTGMCTGAGATGCATGAGCTGCATAAGAGTAAAACMTTGGCTCAAATTACAGGTTTAGAGGCTGCTGTGGCAGGGCTGGAACTGGAGCTGGAATCRTTGKGAGYTCAGAAAYAAGATATAGAYGTTGAGATTGAGAAGAAAGAAACTCAAGTGAAACAACTAGCKGAAGAMAATGRGGGATTGCAAGCCCYAARTTCAGAACTTGWATCAATATCAAATGAGAGAGAAGCTGAACTTTCTGCTCTSACSAAYAAACTTGAGGASAMCAYTAYTGAATGGCTAGKACTGGAGCTGGAATCTTTGCGACATGAGAAYAGWGYTWTSGAAGTAGAGATCGAGAYCAAAGAAACTGTAGCAAAACAACTGGGAGAGGAAAATGCGGGACTGCAAGCCCGAATTTCAGAACTTGAATCGACTTTGAAAGACAGAGAAGCTGAACTTTCCGATCTCACGAAGAAACTTGAGGACAGCAATCATGAATCGTCATCCAGAATAGCAGATTTGTCCGCACAGATCAATAATCTGCTAGCTGAGGTAGATTCTTTGCGTGCCCAGAAAGTTGAGTTGGAGGAACTTATAGTATCCAAAGGTGATGAAGCATCGACTCAGGTCAAGGGATTAACGGAACAAGTAAATGTGTTGCAGCAGGAATTGTTGTCAATGCAGAGCGGGAAAACTGAATTGCAAGTGCAGCTTGAGAACAAAACTCAAGAAATAACGGATCATGAGAGGGTTGTGGAAGAGAAAGAGAGTTTGACGGCAGAAAAGAGAGAGATTGAGATAAAGGTGGACTCAATACACAACCACAAAAGTGAACTCGAAGAGGAGATAAGAACAAAATGCCTCGAGAGTGATCAATTGAGAGTGGAAATTGTTGAGCTAAGGGATCAAATTGTTGAATTTGAGAGGAGACTAACAGAAAAAGAGGCCGAGTTTTCTTCCCTCCAGGAGAAACATGACAGTGCAGTGAATGACACTTCTGCTCAAATAACAGCCTTTGTATCACAGGTTACTAATCTACAACAGGATTTGGATTCATTGCAGGCTGAGAAGAACCAGATGGAGTTGCAGTTTGAGAGGGAGAAACAAGAACTTTCACAGAGCCTGACGCAATTGGAAAATGAAAAGGTTGAGTTAGAGAGCAAGATTGCTGATCATCAGAGACTGCTGAATGAACACGAGGAGACATATGGCAAGTTAAAAGATGAATATATGCAGCTGGAGAGTCATATCCAGGACAGTAAGGTCAACCAAGACGCTGCAGAAAGGAAAATCGAGCAAATGGCGGAAGATTTCAGTAAGAAAATTGAATCCAAAGATGAGACAATAGCTGATTTGGAGCAAGAGGCTGAATATCTGAAACGAGATCTTGAAGAAAAAGGGTACGAGCTTAGTTCTTTGGTTGAAAACTCCCGTAATGTTGAAGTTAAGCTCCGCCTGTCAAACCAGAAGCTCCGGGTTACAGAGCAGGTATTGACTGAGAAAGAGGAGAGCTTCAGAAAGGCAGAACTGAAATTCCTGGAAGAACAGAGAGCACTTGAAGACAGGATTGCTAGATTGTCTGATATAATCTCTGCAAACAACGAAGCCTATCAAAGAAACATCACACTTGTTGCAGAAAATGTGAACAGTTATTTCAGCGGAATGGAATCCATGATAAAAAAATTTATGGACGACTGTGCAGAGTATGAGACGTGCATTCTGGAAACATCGCAGGAGCTTCACGTTGCAAAGAATTGGGTTGCAGAAACAAGGAGTGAAAGAGAGACGTTGAAGAGGGAGGTGGGGGACCTAATTGAACAACTGCGAGATAAGAAAGAAGAAGCATTGGTGCTTGGAGAGCAGGTCGAGAGGATGCGGGCAAAGGCAAGCAAGGAAGAAGTGGAGAAGGGGAGTCTGATCAAGGCCATGAGCCAACTTGAGAAGAAAGCGGCGGACTTGGAGAAGGTGGTGGAGGAGAAAACAGAGGGAATGCTGGGATTAGGAGAGGAGAAGAGGGAAGCCATAAGACAGTTGTGCATATGGATCGAGTATCACCAAAGCCGGTACGATCATCTCAAGGAAATTCTCTCGAAGACAACTCCTGCAAGAGGCCAGACGAGGGCTTCGACATCTCGTCCTTGA

The protein sequence of MdCIP1

MPKHRMREIKSLFGSHINSQKHEELKGTKIGIEEKVNKILKLLKNEELKKDDIPVENSKVEPLAELIQDFHKDYQSLYAQYDQLTGALKEKVWSKQEKDNSSSSSSDSDSDHSSNEKSRKNGVMESDFQKITDGIKQELELAHQEVADLKRRLTATSEEKEALSSDCAAALTKIEEKESVFIDLKTAAERLDAEKSLLLAENGELKQKLEAGGKIEAELIQKVEDVEGEKDNLIKEKETALRTIEDGETITAELRTVIDRLKDEKVTLEQELESVRGEVIHIKQKLQSAEEQVSDLSHNLKAKEEETTESSQLREKLGQREVEYLALSEMHELHEKETLAQIMELQAAVTGLELELESLRAQKRDMEVKIESTEAEVKQLGEVSAGLQVQISEVESISNEKAAELSALTKNLEDYKSESIQLKEKLGQREVECSALSEMHELHKSKTLAQITGLEAAVAGLELELESLRGQKTDIEVEIENKETQVKQLAEENAGLQARISQLESISNEREAEFSSLAKKFEDSSNEYGQLQEKLGQREVEHSTLSEMHELHKSKTLAQITGLEAAVAGLELELESLRDQKKDIEVEIEKKETQVKQLAEENAGLQAQTSELESISNEREAEXSALTXKLXEINXESIQXKEQLDQKEKEYSALSEKHELHESKTSAQILGLEAAVAGLELELRSLRGQKEDIDVEIENKETQVKQLAEENVGLQARISQLESISNEREAEFSSLXKKFEDSSNEYGRLQEKLGQREVEHSTLSEMHELHKSKTLAQITGLEAAVAGLELELESLRXQKKDIEVEIEKKETQVKQLAEXNAGLQAQTSELVSISNEREAEXSALTXKLEEINSESIQLKEXLDIXVEIENKETQVKQLAEENXGLQARISQLESISNEREAEFSSLAKKFEDSSNEYGXLQEKLGQXEVEHSTLXEMHELHKSKTLAQITGLEAAVAGLELELESLRXQKXDIEVEIEKKETQVKQLAEXNXGLQAXXSELXSISNEREAELSALTKKLEXXXXEWLXLELESLRHEKXXXEVEIEXKETVAKQLGEENAGLQARISELESTLKDREAELSDLTKKLEDSNHESSSRIADLSAQINNLLAEVDSLRAQKVELEELIVSKGDEASTQVKGLTEQVNVLQQELLSMQSGKTELQVQLENKTQEITDHERVVEEKESLTAEKREIEIKVDSIHNHKSELEEEIRTKCLESDQLRVEIVELRDQIVEFERRLTEKEAEFSSLQEKHDSAVNDTSAQITAFVSQVTNLQQDLDSLQAEKNQMELQFEREKQELSQSLTQLENEKVELESKIADHQRLLNEHEETYGKLKDEYMQLESHIQDSKVNQDAAERKIEQMAEDFSKKIESKDETIADLEQEAEYLKRDLEEKGYELSSLVENSRNVEVKLRLSNQKLRVTEQVLTEKEESFRKAELKFLEEQRALEDRIARLSDIISANNEAYQRNITLVAENVNSYFSGMESMIKKFMDDCAEYETCILETSQELHVAKNWVAETRSERETLKREVGDLIEQLRDKKEEALVLGEQVERMRAKASKEEVEKGSLIKAMSQLEKKAADLEKVVEEKTEGMLGLGEEKREAIRQLCIWIEYHQSRYDHLKEILSKTTPARGQTRASTSRP

The coding sequence of *MdCOP1*

ATGCCTGCAGGTCGACGATTAGAAAGAATGGAGGAGTGCTCGACCGGGGCTCTAGTCCCGGCAGTGAAACCCGAACCAAAAGCGTCAAGCATCACGGATTTGGCAGATCCAGGTAGCGGTGAGGTGGGTCGTCTGGTGCATGAGAAAGAACTGGCAGAGGTAGACAAGGACTTGTTATGCCCAATTTGTATGCAAATGATAAAGGACGCCTTCCTCACAGCGTGTGGTCATAGCTTCTGCTACATGTGCATCATCACCCACCTCCGCAACAAGAGCGACTGCCCTTGCTGCGCCCAGTTCCTCAGCGCCAAACAATTGTTCCCTAATTTTTTGCTGGACAAGCTTCTGAAGAAGACTTCTGCTCGTCAAATTTCCAAAAGTGCATCTCCTGTAGAGCATGTTCGCCAGGCATTGCACCAGGGATGTGAAGTGTCAATCAAGGAGCTAGACACCCTGTTGGCACTCCTGGCAGAGAAGAAGAGGAAAATGGAACAAGAAGAGGCGGAGAGAAACATGCAAATACTGCTTGACTTCTTGAATTGCCTAAGGAAGCAAAAAGTTGACGAGCTTAATGAGGTGCAAACCCATCTCCAATTTATCAAAGAGGACATAGGTGCAGTAGAGAGACGTGGAATGGAGTTATACCGTGCCAGGGACAAATACTCTGTCAAGCTGCGGATGCTTGGAGCGGATGATTCTATTTATGGGGCAAGAAAGCAATGGCATTCCTCTACAGATAACAATACCGGTGCACTTAGTGGACGAGGAGGGATGTCAATTTGGAATCTTCCAAGAAAGGATGGATCAGACTCCCAGTATATGACTCAAACTGGTCTTGCCATAGCCAGAAAAAAGCGGGTCCATGCACAGTTTGGTGAACTCCAAGAGTGTTACCTGCAAAAGCGGCATCAGATGGTGAACCAACCATATTCCCAGCAAGAACAGGACAAAGGTGTAATACAAAGAGAAGGTTATACTGCAGGTCTTGCTGATTTTCAAACAGTGCTCACTACGTTAACACGTTACAGTCGAATGAGGGTCATTGCTGAACTTAGGCATGGGGATCTATTTCACTCAGCCAATATAGTATCCAGCATTGAATTTGACTGTGATTATGAGTTATTTGCTACTGCCGGAGTATCACGGTGCATAAAAGTTTTTGACTTCTCTTCGGTTTTGAATGATCCAGCTGACGTGCACTGTCCTGTTGTGGAGATGCCTACACGTTCAAAGCTTAGTTGCTTGAGCTGGAACAAGTTTACTAAAAACCATATAGCCAGTACTGATTATGAGGGAATAGTAACAGTTTGGGATGTAAATACTCGGCAGAGTATCATGGAATATGAAGAGCATGAAAAACGTGCTTGGAGTGTTGATTTTTCATGCACAGAGCCCACAAGGCTTGTATCTGGTAGTGATGATTGTAAGTTAAAACTTTGGTGCACAAGGCAGGAAGCTAGCGTTATGGATATTGACATGAAAGCCAACATATGTTCCGTCAAGTATAATCCAGGATCTGGAAACTGCATTGCGGTTGGTTCAGCAGACCATCACATCCACTATTATGATTTAAGAAATCCGAGCGAACCACTCCATGTGTTCACTGGGCATGGGAAAGCTGTTTCTTATGTAAAGTTCTTGTCAAACTATGAGCTTGCCTCTGCATCCACTGATAGCACATTGCGGTTATGGAATGTGAGGGATAATATTCCAGTTCGTACTTTCAAAGGTCACACAAATGAGAAGAACTTTGTAGGTCTTACAGTAAACAGCGAATACATTGCATGTGGCAGCGAAACAAATGAAGTGTTCGTGTATCATAAGGAAATCTCTAAACCGGTGACTTGGCATAAGTTTGGGTCGCCTGATTTGGATGACGCTGATGATGATGCGGGGTCGTACTTCATCAGTGCTGTATGTTGGAAGAGTGATGGCCCTACCATGCTAACTGCTAACAGTCAAGGAACCATTAAAGTGTTGGTTCTTGCGGCATAG

The protein sequence of MdCOP1

MPAGRRLERMEECSTGALVPAVKPEPKASSITDLADPGSGEVGRLVHEKELAEVDKDLLCPICMQMIKDAFLTACGHSFCYMCIITHLRNKSDCPCCAQFLSAKQLFPNFLLDKLLKKTSARQISKSASPVEHVRQALHQGCEVSIKELDTLLALLAEKKRKMEQEEAERNMQILLDFLNCLRKQKVDELNEVQTHLQFIKEDIGAVERRGMELYRARDKYSVKLRMLGADDSIYGARKQWHSSTDNNTGALSGRGGMSIWNLPRKDGSDSQYMTQTGLAIARKKRVHAQFGELQECYLQKRHQMVNQPYSQQEQDKGVIQREGYTAGLADFQTVLTTLTRYSRMRVIAELRHGDLFHSANIVSSIEFDCDYELFATAGVSRCIKVFDFSSVLNDPADVHCPVVEMPTRSKLSCLSWNKFTKNHIASTDYEGIVTVWDVNTRQSIMEYEEHEKRAWSVDFSCTEPTRLVSGSDDCKLKLWCTRQEASVMDIDMKANICSVKYNPGSGNCIAVGSADHHIHYYDLRNPSEPLHVFTGHGKAVSYVKFLSNYELASASTDSTLRLWNVRDNIPVRTFKGHTNEKNFVGLTVNSEYIACGSETNEVFVYHKEISKPVTWHKFGSPDLDDADDDAGSYFISAVCWKSDGPTMLTANSQGTIKVLVLAA

The coding sequence of *AtCIP1*

ATGAAAAAGCATAAGTTTAGAGAAACCTTGAAGTCTTTCTTTGAGCCTCATTTTGATCATGAGAAAGGTGAAATGCTTAAAGGAACTAAAACTGAAATAGATGAAAAGGTGAACAAAATCTTGGGAATGGTTGAGAGTGGAGACGTCAATGAAGATGAGTCCAATAGACAAGTGGTTGCAGACTTAGTGAAGGAATTCTACAGCGAATACCAGTCCCTGTACCGCCAGTACGATGATCTAACTGGAGAGATTAGGAAAAAGGTCAACGGGAAAGGAGAAAGCTCCTCTTCATCAAGCTCAGACTCGGATTCTGACCATTCTTCTAAGAGGAAGGTCAAGAGAAATGGAAATGGAAAAGTAGAGAAAGATGTAGAGTTGGTAACAGGTGCCCTGAAGCAACAAATCGAAGCTGCAAATCTTGAAATTGCTGATCTGAAAGGGAAGTTGACAACGACTGTTGAAGAAAAAGAAGCAGTAGATTCTGAGCTTGAATTAGCTTTGATGAAGTTAAAAGAATCAGAAGAGATTAGCAGTAAGTTGAAACTTGAAACTGAGAAGTTAGAGGACGAAAAGTCAATAGCACTGAGTGATAACAGGGAACTGCATCAGAAACTGGAAGTTGCTGGCAAAACAGAAACTGATCTGAACCAGAAGTTAGAAGATATAAAAAAAGAGAGAGATGAACTGCAAACTGAGAGGGACAATGGTATCAAAAGATTTCAAGAAGCTGAAAAAGTTGCAGAAGATTGGAAAACAACGAGTGATCAGCTCAAAGATGAAACTTCTAATCTCAAGCAGCAGCTTGAAGCATCAGAGCAGCGAGTTTCAGAGCTGACCAGCGGTATGAATAGTGCAGAGGAAGAGAACAAATCTCTATCCTTGAAAGTTTCGGAGATTTCAGATGTGATCCAACAGGGACAGACCACCATACAAGAACTAATTTCCGAATTGGGAGAGATGAAGGAAAAGTACAAGGAAAAAGAGAGTGAGCATTCTAGTTTGGTGGAGTTACATAAGACCCATGAGAGAGAATCATCAAGTCAGGTGAAAGAATTAGAAGCACACATAGAATCATCAGAGAAGTTGGTTGCAGATTTCACCCAAAGCCTGAACAATGCAGAGGAAGAGAAAAAACTGCTATCTCAGAAAATAGCAGAACTCTCTAACGAGATTCAAGAGGCGCAGAACACCATGCAAGAACTCATGTCTGAGTCTGGGCAGTTGAAAGAGAGCCACAGTGTGAAAGAGCGAGAACTTTTCAGTTTGAGGGACATCCATGAGATTCATCAAAGAGACTCATCCACCAGAGCAAGTGAATTAGAAGCTCAACTGGAGTCCTCAAAACAGCAGGTCTCAGATTTGAGTGCGAGTCTGAAAGCTGCAGAGGAAGAAAACAAAGCTATATCCTCGAAAAACGTGGAAACTATGAACAAACTCGAACAAACGCAGAACACGATACAGGAACTCATGGCTGAATTGGGAAAGTTGAAAGACAGCCACAGAGAGAAAGAGAGTGAGCTTTCTAGTTTGGTGGAAGTACACGAGACTCACCAGAGAGATTCATCAATTCATGTGAAAGAATTAGAAGAGCAAGTGGAATCATCAAAGAAATTGGTTGCGGAGTTGAACCAAACCCTGAACAATGCAGAGGAAGAGAAAAAAGTGCTATCTCAGAAAATAGCAGAACTTTCTAACGAGATTAAAGAGGCACAAAACACCATACAAGAACTCGTGTCTGAGTCTGGGCAGTTGAAAGAGAGCCACAGTGTAAAGGATAGAGATCTTTTCAGCTTGAGGGACATCCACGAGACTCATCAAAGAGAATCATCCACTCGCGTGAGTGAATTAGAAGCTCAACTGGAATCCTCAGAACAGCGGATCTCAGATTTGACTGTGGATCTGAAGGATGCAGAGGAAGAAAACAAAGCTATCTCCTCGAAAAATTTGGAAATTATGGACAAGCTTGAACAGGCTCAGAACACGATAAAAGAACTCATGGATGAATTGGGAGAGTTGAAAGACCGACACAAAGAGAAAGAGAGTGAGCTTTCTAGTTTGGTGAAGTCAGCAGATCAACAGGTTGCAGATATGAAGCAGAGTCTGGACAATGCAGAAGAAGAGAAAAAAATGTTATCTCAGAGAATCTTAGATATCTCTAATGAGATTCAAGAAGCACAAAAAACCATACAAGAACACATGTCTGAGTCTGAACAGTTGAAAGAGAGCCACGGTGTGAAAGAGAGGGAACTTACTGGTTTGAGGGACATTCACGAGACTCATCAAAGAGAATCATCCACTCGTTTGAGTGAATTAGAAACTCAACTGAAATTATTAGAACAACGGGTCGTAGATCTGAGTGCGAGTCTGAATGCTGCAGAGGAAGAAAAGAAGTCCCTGTCCTCAATGATCTTGGAAATTACGGATGAGCTCAAACAGGCCCAAAGCAAGGTACAGGAACTTGTGACTGAATTGGCAGAGTCTAAAGATACACTCACACAAAAAGAGAATGAGCTTTCTAGTTTTGTGGAGGTACACGAGGCCCATAAGAGAGATTCCTCAAGTCAGGTGAAAGAATTAGAAGCACGGGTGGAATCAGCAGAGGAACAGGTTAAAGAATTGAACCAGAACCTGAACAGTTCAGAGGAAGAGAAGAAAATTTTATCTCAGCAAATTTCAGAAATGTCGATCAAGATCAAGCGGGCGGAAAGCACCATACAAGAACTCAGCTCTGAGTCTGAACGATTAAAAGGGAGCCACGCTGAGAAAGACAATGAACTTTTTAGCTTGAGGGATATCCATGAGACTCATCAGAGAGAATTATCCACTCAGTTAAGAGGTTTAGAAGCGCAACTGGAATCATCTGAACACAGGGTTTTGGAATTGAGCGAAAGTCTGAAGGCTGCAGAAGAAGAAAGCAGAACTATGTCCACGAAAATCTCAGAAACTTCAGATGAGCTTGAACGGACACAGATCATGGTACAGGAACTCACAGCTGATTCGAGCAAACTGAAAGAGCAGCTCGCTGAGAAAGAAAGCAAACTATTCCTTCTGACAGAGAAGGACAGCAAATCACAGGTGCAAATAAAAGAACTAGAAGCAACAGTAGCGACACTGGAGCTGGAACTAGAGTCAGTTCGTGCCCGTATAATAGATCTTGAGACAGAGATTGCAAGCAAGACCACCGTAGTTGAGCAGTTGGAAGCGCAAAACAGAGAAATGGTTGCTAGAATCTCAGAACTTGAGAAGACAATGGAGGAGAGAGGAACTGAACTCTCAGCTTTAACTCAAAAACTTGAGGATAACGATAAGCAATCATCGTCTTCAATTGAGACTTTGACAGCTGAGATCGATGGCCTACGAGCAGAATTAGATTCAATGTCTGTTCAAAAAGAAGAGGTGGAGAAACAAATGGTGTGCAAAAGCGAGGAAGCCTCAGTGAAGATTAAACGTTTGGATGATGAGGTCAATGGTCTGAGACAGCAAGTGGCCTCACTTGATAGCCAGAGAGCAGAACTCGAGATCCAACTTGAAAAGAAGTCCGAGGAGATATCTGAATATCTGAGTCAGATTACAAATCTAAAAGAGGAGATCATAAACAAGGTTAAAGTTCACGAGAGTATTCTAGAAGAAATAAATGGTTTATCTGAGAAGATTAAGGGTCGTGAACTTGAGTTAGAGACTCTAGGGAAACAGAGAAGTGAGCTTGATGAGGAGCTGAGAACTAAGAAAGAAGAGAATGTTCAAATGCACGATAAGATCAACGTAGCGTCTTCTGAAATAATGGCCTTAACAGAACTGATCAACAATCTGAAGAATGAGCTTGATTCTCTACAGGTGCAGAAGAGCGAAACCGAAGCAGAGCTTGAGAGAGAGAAGCAAGAGAAATCAGAATTGTCGAATCAGATCACCGATGTCCAGAAAGCATTGGTAGAGCAAGAAGCTGCTTACAATACGCTGGAAGAGGAACACAAGCAGATAAACGAACTATTTAAAGAAACTGAAGCAACACTAAATAAGGTAACTGTGGATTACAAAGAAGCTCAAAGATTGTTGGAGGAGAGGGGTAAGGAAGTGACATCCAGAGATTCTACAATTGGGGTTCATGAAGAGACGATGGAGAGTTTACGTAACGAGCTGGAAATGAAAGGAGACGAGATCGAAACTCTCATGGAGAAGATCAGTAACATCGAGGTTAAGCTACGCTTGTCGAACCAGAAACTGAGAGTAACCGAACAGGTACTAACAGAGAAAGAAGAAGCTTTCAGGAAAGAAGAGGCTAAGCACTTAGAGGAGCAAGCATTGCTTGAGAAGAATCTCACCATGACACATGAGACTTATCGAGGTATGATCAAAGAGATAGCAGATAAAGTGAACATAACAGTAGATGGGTTTCAATCCATGTCAGAAAAACTCACGGAGAAACAGGGGAGATACGAGAAAACTGTAATGGAGGCATCAAAAATACTGTGGACTGCGACGAATTGGGTGATAGAGAGAAATCACGAGAAGGAGAAGATGAATAAAGAGATAGAGAAGAAAGATGAAGAAATAAAAAAGCTTGGAGGAAAAGTAAGAGAAGATGAAAAAGAGAAGGAGATGATGAAAGAGACTTTGATGGGACTTGGAGAAGAGAAAAGAGAAGCGATAAGGCAATTATGTGTTTGGATCGATCACCATAGAAGTCGTTGTGAATATCTTGAGGAGGTTTTGTCTAAGACCGTTGTGGCTCGAGGCCAAAGAAGAGTGTCGCAGCGAACTTAA

The protein sequence of AtCIP1

MKKHKFRETLKSFFEPHFDHEKGEMLKGTKTEIDEKVNKILGMVESGDVNEDESNRQVVADLVKEFYSEYQSLYRQYDDLTGEIRKKVNGKGESSSSSSSDSDSDHSSKRKVKRNGNGKVEKDVELVTGALKQQIEAANLEIADLKGKLTTTVEEKEAVDSELELALMKLKESEEISSKLKLETEKLEDEKSIALSDNRELHQKLEVAGKTETDLNQKLEDIKKERDELQTERDNGIKRFQEAEKVAEDWKTTSDQLKDETSNLKQQLEASEQRVSELTSGMNSAEEENKSLSLKVSEISDVIQQGQTTIQELISELGEMKEKYKEKESEHSSLVELHKTHERESSSQVKELEAHIESSEKLVADFTQSLNNAEEEKKLLSQKIAELSNEIQEAQNTMQELMSESGQLKESHSVKERELFSLRDIHEIHQRDSSTRASELEAQLESSKQQVSDLSASLKAAEEENKAISSKNVETMNKLEQTQNTIQELMAELGKLKDSHREKESELSSLVEVHETHQRDSSIHVKELEEQVESSKKLVAELNQTLNNAEEEKKVLSQKIAELSNEIKEAQNTIQELVSESGQLKESHSVKDRDLFSLRDIHETHQRESSTRVSELEAQLESSEQRISDLTVDLKDAEEENKAISSKNLEIMDKLEQAQNTIKELMDELGELKDRHKEKESELSSLVKSADQQVADMKQSLDNAEEEKKMLSQRILDISNEIQEAQKTIQEHMSESEQLKESHGVKERELTGLRDIHETHQRESSTRLSELETQLKLLEQRVVDLSASLNAAEEEKKSLSSMILEITDELKQAQSKVQELVTELAESKDTLTQKENELSSFVEVHEAHKRDSSSQVKELEARVESAEEQVKELNQNLNSSEEEKKILSQQISEMSIKIKRAESTIQELSSESERLKGSHAEKDNELFSLRDIHETHQRELSTQLRGLEAQLESSEHRVLELSESLKAAEEESRTMSTKISETSDELERTQIMVQELTADSSKLKEQLAEKESKLFLLTEKDSKSQVQIKELEATVATLELELESVRARIIDLETEIASKTTVVEQLEAQNREMVARISELEKTMEERGTELSALTQKLEDNDKQSSSSIETLTAEIDGLRAELDSMSVQKEEVEKQMVCKSEEASVKIKRLDDEVNGLRQQVASLDSQRAELEIQLEKKSEEISEYLSQITNLKEEIINKVKVHESILEEINGLSEKIKGRELELETLGKQRSELDEELRTKKEENVQMHDKINVASSEIMALTELINNLKNELDSLQVQKSETEAELEREKQEKSELSNQITDVQKALVEQEAAYNTLEEEHKQINELFKETEATLNKVTVDYKEAQRLLEERGKEVTSRDSTIGVHEETMESLRNELEMKGDEIETLMEKISNIEVKLRLSNQKLRVTEQVLTEKEEAFRKEEAKHLEEQALLEKNLTMTHETYRGMIKEIADKVNITVDGFQSMSEKLTEKQGRYEKTVMEASKILWTATNWVIERNHEKEKMNKEIEKKDEEIKKLGGKVREDEKEKEMMKETLMGLGEEKREAIRQLCVWIDHHRSRCEYLEEVLSKTVVARGQRRVSQRT

The coding sequence of *AtCOP1*

ATGGAAGAGATTTCGACGGATCCGGTTGTTCCAGCGGTGAAACCTGACCCGAGAACATCTTCAGTTGGTGAAGGTGCTAATCGTCATGAAAATGACGACGGAGGAAGCGGCGGTTCTGAGATTGGAGCACCGGATCTGGATAAAGACTTGCTTTGTCCGATTTGTATGCAGATTATTAAAGATGCTTTCCTCACGGCTTGTGGTCATAGTTTCTGCTATATGTGTATCATCACACATCTTAGGAACAAGAGTGATTGTCCCTGTTGTAGCCAACACCTCACCAATAATCAGCTTTACCCTAATTTCTTGCTCGATAAGCTATTGAAGAAAACTTCAGCTCGGCATGTGTCAAAAACTGCATCGCCCTTGGATCAGTTTCGGGAAGCACTACAAAGGGGTTGTGATGTGTCAATTAAGGAGGTTGATAATCTTCTGACACTTCTTGCGGAAAGGAAGAGAAAAATGGAACAGGAAGAAGCTGAGAGGAACATGCAGATACTTTTGGACTTTTTGCATTGTCTAAGGAAGCAAAAAGTTGATGAACTAAATGAGGTGCAAACTGATCTCCAGTATATTAAAGAAGATATAAATGCCGTTGAGAGACATAGAATAGATTTATACCGAGCTAGGGACAGATATTCTGTAAAGTTGCGGATGCTCGGAGATGATCCAAGCACAAGAAATGCATGGCCACATGAGAAGAACCAGATTGGTTTCAACTCCAATTCTCTCAGCATAAGAGGAGGAAATTTTGTAGGCAATTATCAAAACAAAAAGGTAGAGGGGAAGGCACAAGGAAGCTCTCATGGGCTACCAAAGAAGGATGCGCTGAGTGGGTCAGATTCGCAAAGTTTGAATCAGTCAACTGTCTCAATGGCTAGAAAGAAACGGATTCATGCTCAGTTCAATGATTTACAAGAATGTTACCTCCAAAAGCGGCGTCAGTTGGCAGACCAACCAAATAGTAAACAAGAAAATGATAAGAGTGTAGTACGGAGGGAAGGCTATAGCAACGGCCTTGCAGATTTTCAATCTGTGTTGACTACCTTCACTCGCTACAGTCGTCTAAGAGTTATAGCAGAAATCCGGCATGGGGATATATTTCATTCAGCCAACATTGTATCAAGCATAGAGTTTGATCGTGATGATGAGCTGTTTGCCACTGCTGGTGTTTCTAGATGTATAAAGGTTTTTGACTTCTCTTCGGTTGTAAATGAACCAGCAGATATGCAGTGTCCGATTGTGGAGATGTCAACTCGGTCTAAACTTAGTTGCTTGAGTTGGAATAAGCATGAAAAAAATCACATAGCAAGCAGTGATTATGAAGGAATAGTAACAGTGTGGGATGTAACTACTAGGCAGAGTCTTATGGAGTATGAAGAGCACGAAAAACGTGCCTGGAGTGTTGACTTTTCACGAACAGAACCATCAATGCTTGTATCTGGTAGTGACGACTGCAAGGTTAAAGTTTGGTGCACGAGGCAGGAAGCAAGTGTGATTAATATTGATATGAAAGCAAACATATGTTGTGTCAAGTACAATCCTGGCTCAAGCAACTACATTGCGGTCGGATCAGCTGATCATCACATCCATTATTACGATCTAAGAAACATAAGCCAACCACTTCATGTCTTCAGTGGACACAAGAAAGCAGTTTCCTATGTTAAATTTTTGTCCAACAACGAGCTCGCTTCTGCGTCCACAGATAGCACACTACGCTTATGGGATGTCAAAGACAACTTGCCAGTTCGAACATTCAGAGGACATACTAACGAGAAGAACTTTGTGGGTCTCACAGTGAACAGCGAGTATCTCGCCTGTGGAAGCGAGACAAACGAAGTATATGTATATCACAAGGAAATCACGAGACCCGTGACATCGCACAGATTTGGATCGCCAGACATGGACGATGCAGAGGAAGAGGCAGGTTCCTACTTTATTAGTGCGGTTTGCTGGAAGAGTGATAGTCCCACGATGTTGACTGCGAATAGTCAAGGAACCATCAAAGTTCTGGTACTCGCTGCGTGA

The protein sequence of AtCOP1

MEEISTDPVVPAVKPDPRTSSVGEGANRHENDDGGSGGSEIGAPDLDKDLLCPICMQIIKDAFLTACGHSFCYMCIITHLRNKSDCPCCSQHLTNNQLYPNFLLDKLLKKTSARHVSKTASPLDQFREALQRGCDVSIKEVDNLLTLLAERKRKMEQEEAERNMQILLDFLHCLRKQKVDELNEVQTDLQYIKEDINAVERHRIDLYRARDRYSVKLRMLGDDPSTRNAWPHEKNQIGFNSNSLSIRGGNFVGNYQNKKVEGKAQGSSHGLPKKDALSGSDSQSLNQSTVSMARKKRIHAQFNDLQECYLQKRRQLADQPNSKQENDKSVVRREGYSNGLADFQSVLTTFTRYSRLRVIAEIRHGDIFHSANIVSSIEFDRDDELFATAGVSRCIKVFDFSSVVNEPADMQCPIVEMSTRSKLSCLSWNKHEKNHIASSDYEGIVTVWDVTTRQSLMEYEEHEKRAWSVDFSRTEPSMLVSGSDDCKVKVWCTRQEASVINIDMKANICCVKYNPGSSNYIAVGSADHHIHYYDLRNISQPLHVFSGHKKAVSYVKFLSNNELASASTDSTLRLWDVKDNLPVRTFRGHTNEKNFVGLTVNSEYLACGSETNEVYVYHKEITRPVTSHRFGSPDMDDAEEEAGSYFISAVCWKSDSPTMLTANSQGTIKVLVLAA
