# Supplementary material for: The apple MdCOP1-interacting protein 1 negatively regulates hypocotyl elongation and anthocyanin biosynthesis
Source: BMC Plant Biol. 2021 Jan 6;21:15. doi: 10.1186/s12870-020-02789-3 (PMC7789773; doi:10.1186/s12870-020-02789-3)
Supplement: Supplementary file 4 — Additional file 4. The original gel/blot images presented in this study. [file 12870_2020_2789_MOESM4_ESM.pdf]

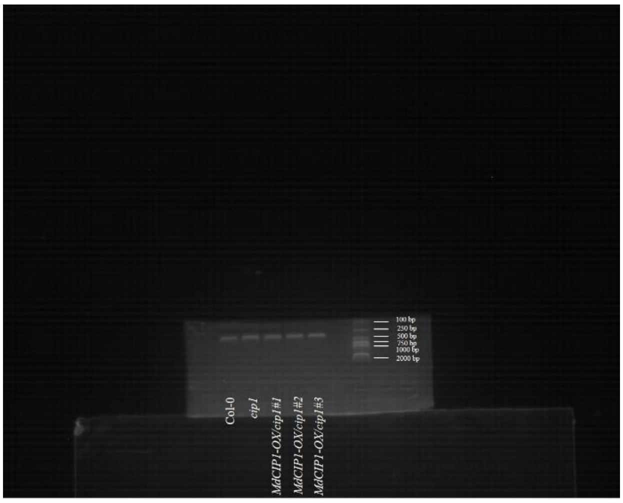

Primers: AtACTIN2-F + AtACTIN2-R, PCR product: 400 bp

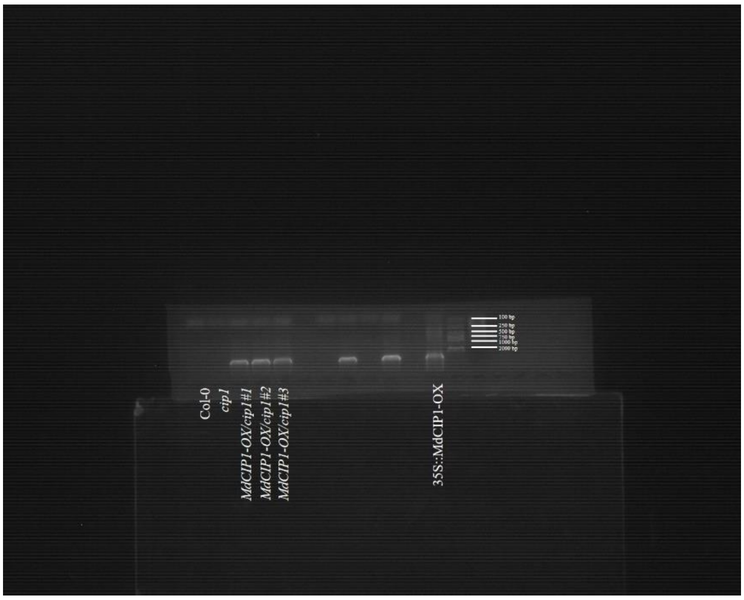

Primers: 35S-F + MdCIP1-R, PCR product: 5.5kb

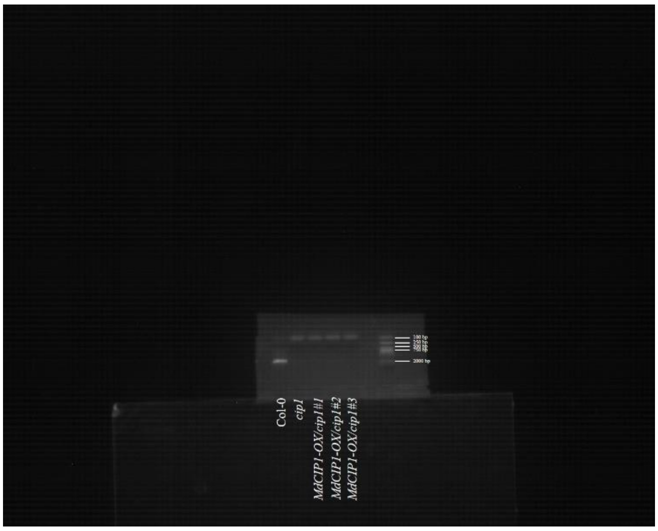

Primers: *cip1*-LP + *cip1*-RP, PCR product: 1150 bp

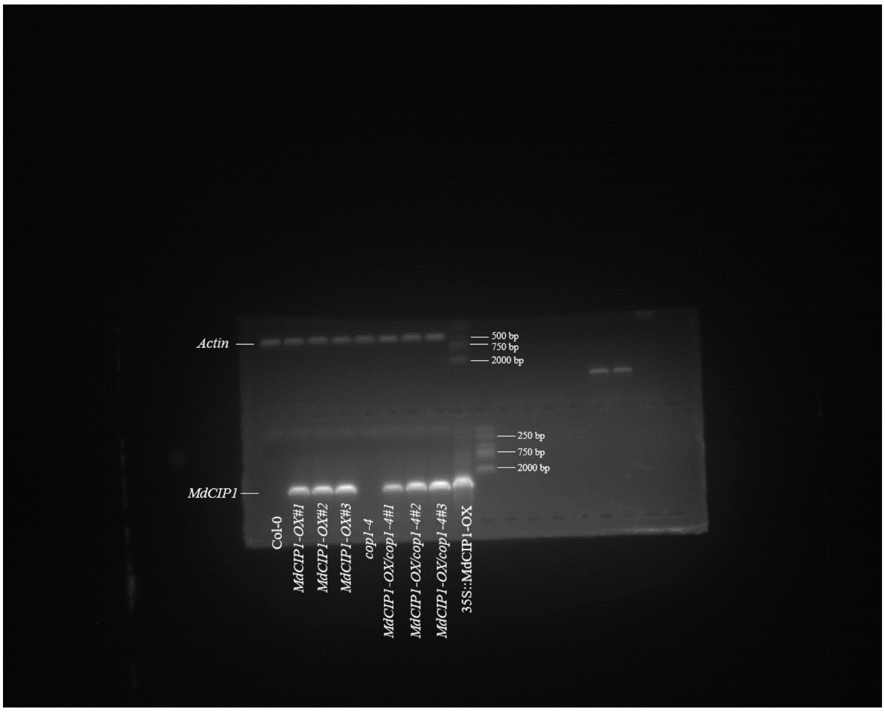

Primers: AtACTIN2-F + AtACTIN2-F, PCR product:400 bp

Primers: 35S-F + MdCIP1-R, PCR product: 5.5kb

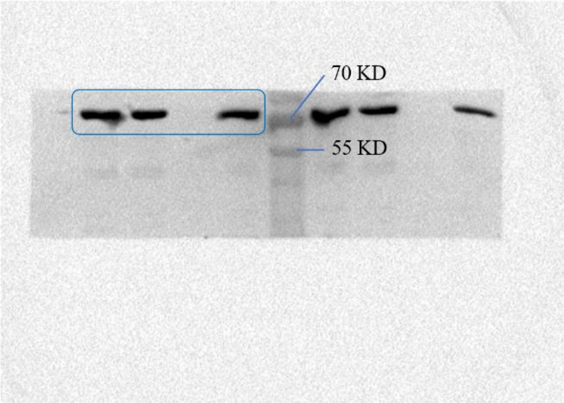

Figure S3 (anti-His)

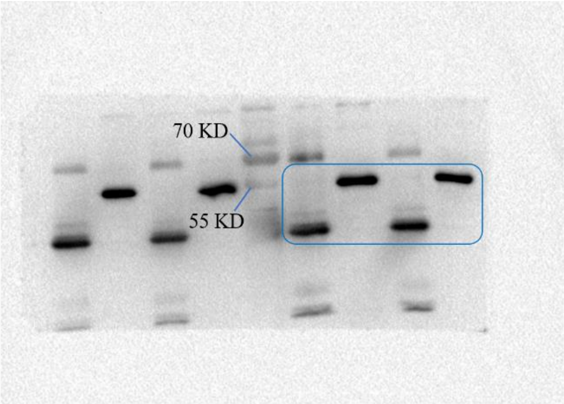

Figure S3 (anti-GST)
