# Supplementary material for: The apple MdCOP1-interacting protein 1 negatively regulates hypocotyl elongation and anthocyanin biosynthesis
Source: BMC Plant Biol. 2021 Jan 6;21:15. doi: 10.1186/s12870-020-02789-3 (PMC7789773; doi:10.1186/s12870-020-02789-3)
Supplement: Supplementary file 5 — Additional file 5: Fig. S1. The protein structure comparison of MdCIP1 and AtCIP1. Fig. S2. Identification of the MdCIP1-OX/cip1 Arabidopsis seedlings at the DNA level. Fig. S3. The coiled-coil region of MdCIP1 interacts with MdCOP1 in vitro pull-down assay. Fig. S4. MdCIP1 interacts with AtCOP1. Fig. S5. Identification of the MdCIP1-OX/cop1–4 Arabidopsis seedlings at the DNA level. Fig. S6. MdCIP1-OX/cop1–4 presents the cop1–4 phenotype at the adult stage. [file 12870_2020_2789_MOESM5_ESM.zip › Figure S1.docx]

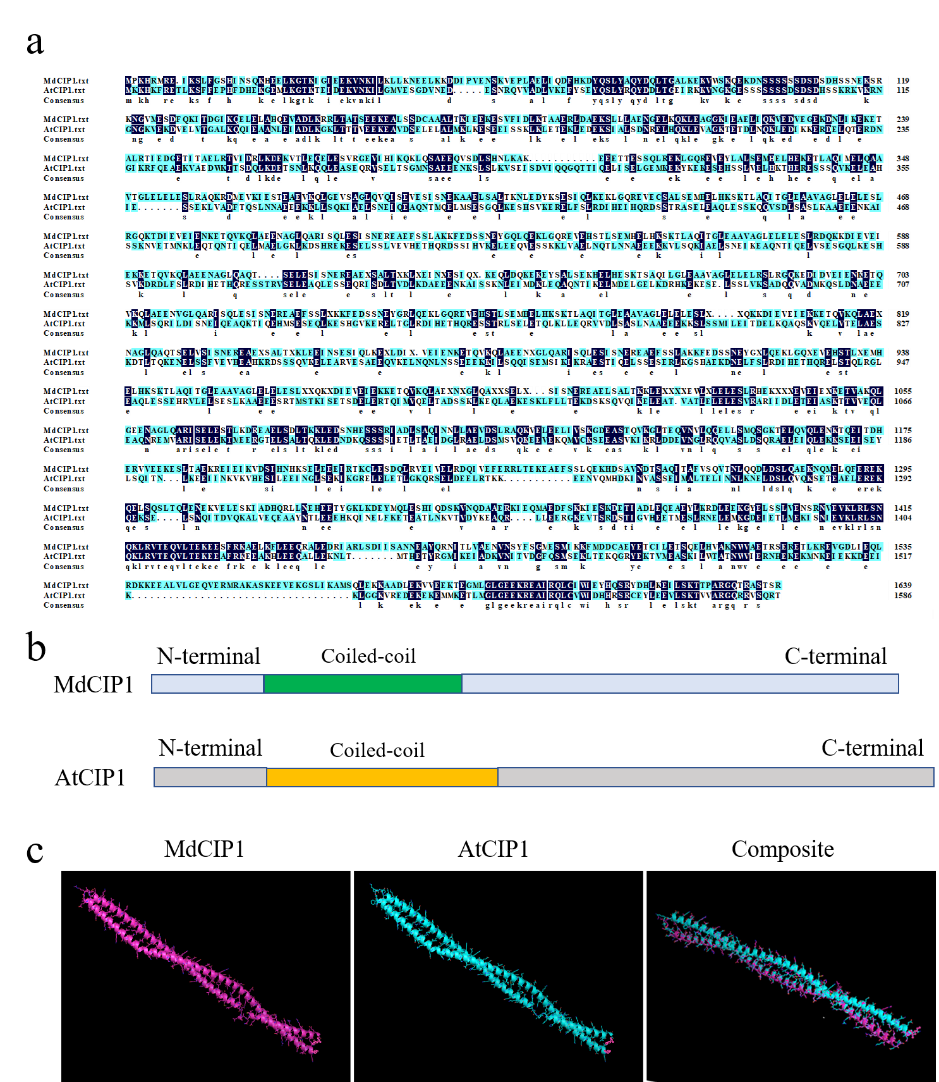


**Figure S1** The protein structure comparison of MdCIP1 and AtCIP1.

(a) the protein primary structure comparison of MdCIP1 and AtCIP1. (b) the protein secondary structure comparison of MdCIP1 and AtCIP1. (c) the protein 3D structure comparison of MdCIP1 and AtCIP1.
