# Supplementary material for: The apple MdCOP1-interacting protein 1 negatively regulates hypocotyl elongation and anthocyanin biosynthesis
Source: BMC Plant Biol. 2021 Jan 6;21:15. doi: 10.1186/s12870-020-02789-3 (PMC7789773; doi:10.1186/s12870-020-02789-3)
Supplement: Supplementary file 5 — Additional file 5: Fig. S1. The protein structure comparison of MdCIP1 and AtCIP1. Fig. S2. Identification of the MdCIP1-OX/cip1 Arabidopsis seedlings at the DNA level. Fig. S3. The coiled-coil region of MdCIP1 interacts with MdCOP1 in vitro pull-down assay. Fig. S4. MdCIP1 interacts with AtCOP1. Fig. S5. Identification of the MdCIP1-OX/cop1–4 Arabidopsis seedlings at the DNA level. Fig. S6. MdCIP1-OX/cop1–4 presents the cop1–4 phenotype at the adult stage. [file 12870_2020_2789_MOESM5_ESM.zip › Figure S4.docx]

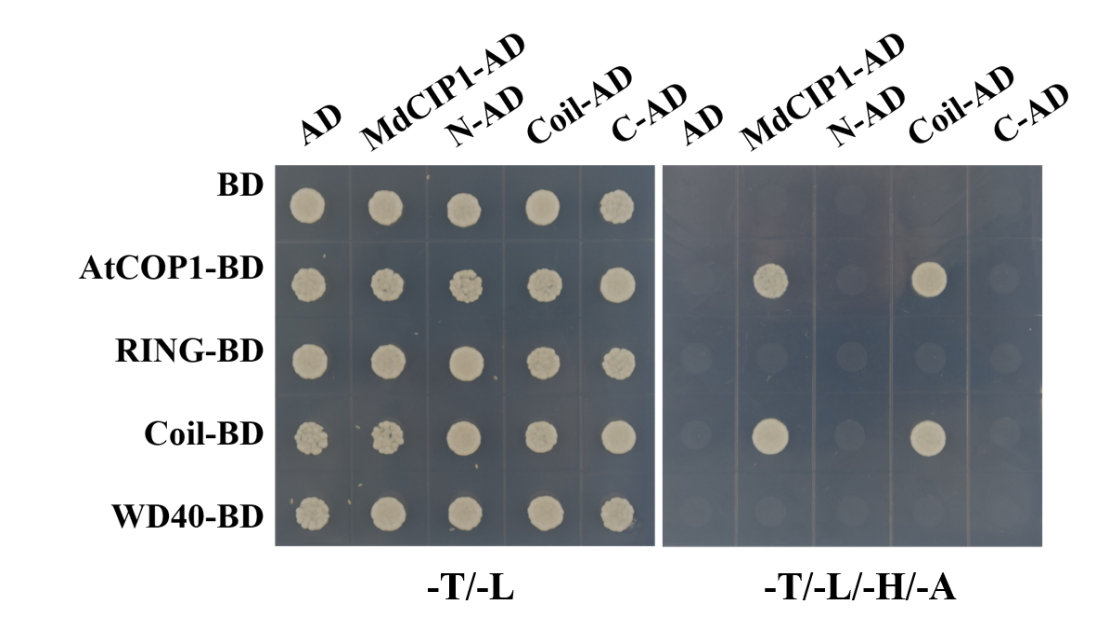


**Figure S4** MdCIP1 interacts with AtCOP1.

The coiled-coil domain of MdCIP1 specifically interacts with the coiled-coil domain of AtCOP1 in Y2H assay.
